# Why variant effect predictors and multiplexed assays agree and disagree

**DOI:** 10.1038/s41467-026-77211-x

**Published:** 2026-08-27

**Authors:** Benjamin J. Livesey, Joseph A. Marsh

**Affiliations:** MRC Human Genetics Unit, Institute of Genetics and Cancer, University of Edinburgh, Edinburgh, UK

**Keywords:** Protein function predictions, Proteomics, Computational models

## Abstract

Multiplexed assays of variant effect (MAVEs) and computational variant effect predictors (VEPs) are two key tools that provide evidence for the interpretation of genetic variants. While their outputs are often concordant, there are also many differences. Here, we analyse missense MAVE data from 40 different human proteins, comparing them to state-of-the-art VEPs in order to quantify and explain their points of agreement and disagreement. We find that discordance is not random but reflects fundamental differences in how each method infers variant effects. VEPs, which rely heavily on sequence conservation and basic structural features, tend to predict buried and bulky hydrophobic residues as more damaging, while underpredicting impact in disordered regions and at charged surface residues. MAVEs, by contrast, capture context-specific mechanisms more accurately, but can miss damaging variants when the assay fails to reflect disease biology, or be subject to high levels of experimental noise. By comparing both global patterns and specific clinically relevant variants, we show how protein features, assay design, and variant type shape prediction discordance. Our findings provide a framework for interpreting when and why MAVEs and VEPs diverge and point toward strategies for improving variant interpretation through integrated, mechanism-aware approaches.

## Abstract (web summary)

Multiplexed assays of variant effect (MAVEs) and computational variant effect predictors (VEPs) are two key tools for the interpretation of genetic variants. Here the authors compare the results from these two approaches and investigate instances where they produce conflicting evidence.

## Introduction

Genetic sequencing is becoming an ever more useful tool for medical diagnosis as advances have continued to make it more accessible and less expensive to perform. Unfortunately, our understanding of the mechanisms underlying the pathogenicity of the numerous novel variants being identified in disease-relevant proteins has not experienced the same amount of progress. This has led to a widening gap between the number of variants observed in sequencing data and the number with assigned clinical interpretations. As of December 2024, 57% of the single nucleotide variants (SNVs) reported in ClinVar^1^ that can be mapped to a UniProt primary isoform^2^ are of uncertain clinical significance, and that percentage is likely to rise as more sequencing is carried out. This also represents only a small fraction of SNVs possible in the sequence space of the protein-coding regions of the human genome.

Assays for protein variant function are a commonly employed tool to provide evidence for variant classification. However, single-variant functional assays are too expensive and time-consuming to adequately fill the gap between the number of novel variants being observed and the need to identify those of clinical significance. To this end, advances have been made in the development and refinement of alternative high-throughput strategies to identify potentially pathogenic variants in the human population.

One such approach is to use multiplexed assays of variant effect (MAVEs)^3^, a general term for a high-throughput, pooled assay in which the effect of a large number of variants on protein function, stability, binding or some other attribute can be assessed. In a typical MAVE for a protein coding gene, a library of variants is created in the gene of interest and then used to transform or transfect cells in which the function of the resultant mutant protein is linked to some measurable attribute such as cellular growth rate or reporter gene expression. The assay used to determine variant effect can be tailored to the specific protein or disease being assessed, for example, DNA binding activity for the PAX6 transcription factor^4^ or protease activity of the caspase proteins, CASP3 and CASP7^5^. If the function of the MAVE target gene is essential for the growth of a given cell line, then the relative growth rate of the variant-bearing cells can be used as a proxy for fitness^6–8^. The relative fitness of each variant can be calibrated by using the measurements of null and wild type variants. While MAVEs can take considerable expertise, time and specialist equipment to carry out, they remain substantially faster and more affordable than performing separate assays for each variant. Several MAVEs have reported extremely good performance for clinical variant classification^9–11^.

Another approach is the use of computational variant effect predictors (VEPs), which aim to estimate the likely functional or clinical impact of genetic variants using features such as sequence conservation, evolutionary patterns, structural context and physicochemical properties^12^. In contrast to wet-lab assays, VEPs are rapid, inexpensive and readily scalable, making them attractive for the interpretation of the large numbers of novel variants emerging from sequencing studies. A wide range of VEPs has now been developed, spanning methods based on sequence alignments, protein language models, structural information and deep learning. These tools are increasingly used to prioritise variants for follow-up and to support clinical interpretation, although their outputs can vary substantially in meaning, performance and accessibility.

There is an important distinction in what MAVEs and VEPs quantify, which raises questions about how directly their scores can be compared. MAVEs typically measure the effect of variants on a specific experimental readout, such as protein activity, stability, or abundance, depending on the assay design. By contrast, VEPs generally estimate a broader notion of variant impact, often derived from evolutionary constraint, sequence context, and structural features. While these scores are frequently used as proxies for pathogenicity, many modern VEPs are not explicitly trained to predict disease, but instead capture more general properties related to fitness or constraint. As a result, similar scores from a MAVE and a VEP do not necessarily reflect the same underlying quantity. For example, a variant may strongly disrupt a particular molecular function without causing disease, or conversely may have near wild-type activity in a given assay but still be pathogenic through mechanisms not captured by that assay. Some MAVEs measure cellular fitness rather than a specific biochemical function^13^, which may align more closely with the signals captured by VEPs, and conversely some predictors aim to estimate more specific properties such as protein stability^14^. Despite these differences, both approaches ultimately attempt to capture the consequences of genetic variation, albeit at different levels and with different assumptions.

Understanding the circumstances in which MAVE and VEP scores are discordant is an important concern for the annotation of variants of uncertain clinical significance (VUS). Both methods are capable of providing complementary evidence under current American College of Medical Genetics and Genomics / Association for Molecular Pathology (ACMG/AMP) guidelines, and can be combined in order to improve evidence strengths^15^. Under these guidelines, VEPs provide at most supporting evidence for pathogenicity, whereas functional assays such as MAVEs can provide up to strong evidence. However, recent recommendations from the ClinGen Sequence Variant Interpretation working group suggest that, under appropriate calibration, VEPs may provide stronger levels of evidence^16^.

In our previous work, we demonstrated a strong correlation between top VEPs and MAVE datasets across numerous proteins^17^, with the best-performing predictors approaching a Spearman’s correlation of 0.8 with some datasets. While both approaches aim to capture the impact of variants, their fundamentally different methodologies are expected to result in discordant predictions in certain proteins and protein regions. Given that a large portion of the evidence for the reclassification of many VUS will likely come from both MAVE and VEP sources, it is important to understand how and why the approaches differ in order to combine their evidence effectively.

In this study, we systematically compare MAVE data from 40 human proteins and 10 representative VEPs to determine the extent and causes of discordance between the scores produced by the different approaches. We identify protein regions in which MAVEs and VEPs tend to differ in their predictions and general properties that may be predictive of such regions in other proteins. Finally, we assess several cases of known pathogenic and benign variants where MAVEs and VEPs produce contradictory results and deduce the reasons for the discrepancies.

## Results

### MAVE and VEP dataset selection

We collected a total of 99 score sets from 47 different MAVE studies measuring the effects of single amino acid substitutions for 40 unique human proteins (Supplementary Data 1), primarily from MAVEdb^18^ and ProteinGym^19^. We only considered MAVEs with scores for at least 1000 variants with a paper or document clearly describing the methodology and for which at least 5 pathogenic or likely pathogenic variants were available in ClinVar. We also excluded datasets originating from extremely large-scale stability studies^20,21^ to avoid over-representation of results generated under similar experimental conditions, particularly protein stability assays. Of the collected MAVE datasets, 48 were generated with a yeast expression system, 35 with human cell lines, 11 with murine cells and 5 with *E. coli*. We stratified the collected MAVEs into five categories based on the characteristics of the selection assay that was performed.

- Activity assays (46) link selection to one particular protein function, or lack of function. For example, assessing transcription factor activity by measuring the level of downstream fluorescent protein produced^22^.
- Fitness assays (19) link selection to growth (or lack of growth) of the cellular expression system without assessing a specific protein function. For example, yeast complementation in the absence of a selective condition^23^.
- Binding assays (17) link selection to the ability of the target protein to bind a specific protein, DNA molecule or other target. For example, yeast one/two hybrid assays^4,24^.
- Stability assays (9) link selection to the expression of the correctly folded target protein. For example, VAMP-seq, where a fluorescent tag is added to the target protein and observed only if the protein folds correctly and is not degraded^25^.
- Trafficking assays (8) link selection to the correct localisation of the target protein. For example, cell-surface trafficking assessed by fluorescence^26^.

Saturation genome editing (SGE) is a cutting-edge technology that allows a variant to be engineered into the native gene locus, and subsequently expressed in its native context. In contrast, most other MAVEs overexpress the protein from a plasmid or similar construct. Our dataset contains 8 SGE MAVE datasets for 7 proteins, falling into both the activity and fitness assay categories (Supplementary Data 1).

The number of missense VEPs released to date is now in the hundreds^12,27^, with our recent study assessing the performance of 97 different methods^17^. Rather than consider all of these here, we selected the 5 top-performing methods from each of two previously defined categories.

Most VEPs belong to the clinical-trained class, having been directly trained on clinical labels (i.e., known pathogenic and benign variants). While these tend to perform well in clinical variant benchmarks, they are also the most vulnerable to data circularity, where overlap or indirect information sharing between training and evaluation datasets can lead to inflated performance estimates^28^. The clinical-trained group in this analysis is composed of: VARITY R, a predictor notable for its strong performance in our benchmark intended to minimise the impact of data circularity^17^. VARITY R is trained using rare variants from both clinical databases and functional assays^29^. mvPPT (missense variant Pathogenicity Prediction Tool) uses gradient boosted decision trees to combine other predictors and is trained using data from ClinVar, the Human Gene Mutation Database (HGMD), gnomAD and UniProt^30^. SNPred is a particularly large meta-predictor, using 33 other predictors as features and is trained against ClinVar data^31^. MetaRNN uses recurrent neural networks to combine several features from the database for Nonsynonymous SNPs’ Functional Predictions (dbNSFP)^32^, and is trained against ClinVar data^33^. MutFormer is a protein language model (PLM) trained with common genetic variants and subsequently tuned with disease mutations^34^.

In contrast, population-free VEPs make no use of human population data either as training labels or features, instead being based on features like evolutionary conservation or protein structure. In practice, this category overlaps strongly with what has often been referred to as unsupervised VEPs. Population-free VEPs are therefore largely immune to data circularity when evaluated against human clinical variants, allowing the full set of known pathogenic and benign variants to be used to relate predictor scores to clinical interpretation. This is particularly important for approaches that aim to translate variant effect scores into quantitative evidence for pathogenicity, as gene-level calibration is fundamentally limited by the number of available labelled variants, and any restriction of the usable variant set can substantially reduce its reliability^35^. To represent population-free VEPs, we used: CPT-1, a VEP composed of two other predictors (EVE, ESM-1v) combined with further structural and conservation data and trained by logistic regression on several functional datasets^36^; GEMME, a relatively simple but highly performant predictor that bases its predictions purely on an MSA^37^; ESCOTT, based on GEMME but adds structural features derived from the PDB^38^; ESM-1v, an ensemble of 5 PLMs specifically constructed for zero-shot prediction of variant effects^39^; and SaProt, a PLM that encodes structural context along with protein sequence^40^.

VEPs of all types tend to rely, to varying degrees, on shared sources of information, particularly evolutionary constraints, and as a result often show substantial agreement with one another^41^. Consistent with this, the VEPs selected for this study are strongly correlated (Supplementary Fig. 1 and Supplementary Data 2), with a mean Spearman’s *ρ* of 0.84. Correlations are even higher within the clinical-trained (*ρ* = 0.88) and population-free (*ρ* = 0.85) groups. In contrast, the average correlation between MAVEs and VEPs is markedly lower (*ρ* = 0.43). For the 18 proteins where multiple MAVEs were available, the mean correlation between assays was 0.61 (Supplementary Data 3). Together, these results indicate that VEPs produce substantially more similar scores to one another than to MAVE measurements, consistent with shared underlying features and assumptions. We note that some commonly used clinical meta-predictors such as REVEL and BayesDel are absent from this analysis. Given the strong inter-VEP correlation, the diverse clinical-trained set should still provide representative coverage.

**Fig. 1:**
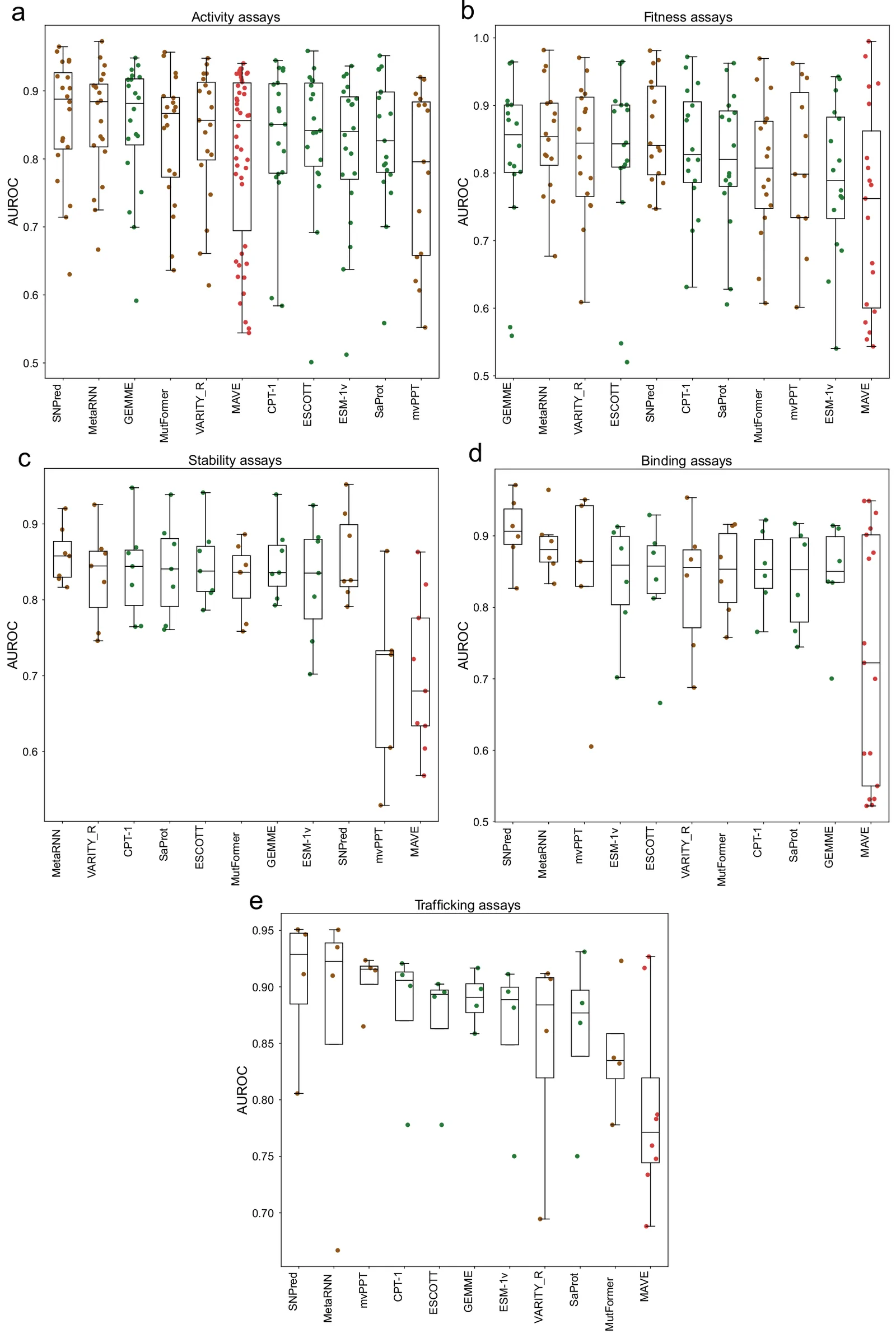
MAVEs and VEPs have heterogeneous performance levels across different proteins. The receiver operating characteristic (ROC) area under the curve (AUC) classification performance of MAVEs, clinically trained VEPs and population-free VEPs for distinguishing pathogenic variants from putatively benign variants, subdivided by MAVE assay type. Population-free VEPs are indicated by green points, clinically trained by brown and MAVEs by red. Note that all MAVEs with sufficient coverage for ROC calculation, which we define as at least 10 pathogenic and 10 putatively benign variants, are included in the MAVE boxplots (denoted as *n* = x), while the VEP boxplots have only 1 result per protein with the corresponding assay type (denoted as *p* = x). **a** Activity assays (*n* = 46, *p* = 20). **b** Fitness assays (*n* = 19, *p* = 16). **c** Stability assays (*n* = 9, *p* = 7). **d** Binding assays (*n* = 17, *p* = 6). **e** Trafficking assays (*n* = 8, *p* = 4). The centre line denotes the median, the box denotes the 25th and 75th percentiles. The whiskers denote the furthest point within 1.5x the interquartile range. Source data for the boxplots are provided in the Source Data file.

We also assessed the performance of MAVE and VEP datasets for clinical variant classification, distinguishing known pathogenic from putatively benign missense variants using the area under the receiver operating characteristic curve (AUROC). Consistent with previous observations^42,43^, AUROC varies widely across proteins (Fig. 1). MAVE performance varies substantially and depends strongly on assay type. For activity assays (Fig. 1a), MAVEs show performance comparable to leading VEPs. However, for fitness, binding, stability and trafficking assays (Fig. 1b–e), the median AUROC of MAVEs is lower than for all VEPs. It is also notable that, for most assay types, the spread of AUROC values is greater for MAVEs, with some datasets showing very high and others very low performance. Together, these results indicate that the performance of MAVEs for clinical classification is highly variable and strongly dependent on the experimental assay.

### Normalisation and comparison of MAVE and VEP scores

The VEP and MAVE datasets are on different scales, making direct comparison difficult. To address this, we first aligned the directionality of all datasets by comparing their Spearman’s correlation to a reference predictor (SIFT) and inverting scores with positive correlation by subtracting them from one. We then normalised each score set independently by converting values to z-scores, preserving the shape of the distributions while enabling direct comparison. Higher z-scores correspond to more damaging predictions or measurements.

For subsequent analyses, we considered VEP-MAVE concordance using VEPs collectively rather than individually. Specifically, we averaged z-scores across the selected VEPs within each group (population-free and clinical-trained) to obtain a single consensus score per variant. Our aim is not to resolve fine-grained differences between individual predictors, but to characterise broader systematic differences between computational predictions and experimental measurements. Aggregating across top-performing VEPs reduces method-specific noise and highlights the shared signal across predictors. We retained separate analyses for clinical-trained and population-free VEPs, both because of their differing susceptibility to data circularity and because clinical-trained predictors provide more limited coverage, typically scoring only amino acid substitutions accessible via single nucleotide variants, whereas the population-free methods in our set score all possible substitutions.

**Fig. 2:**
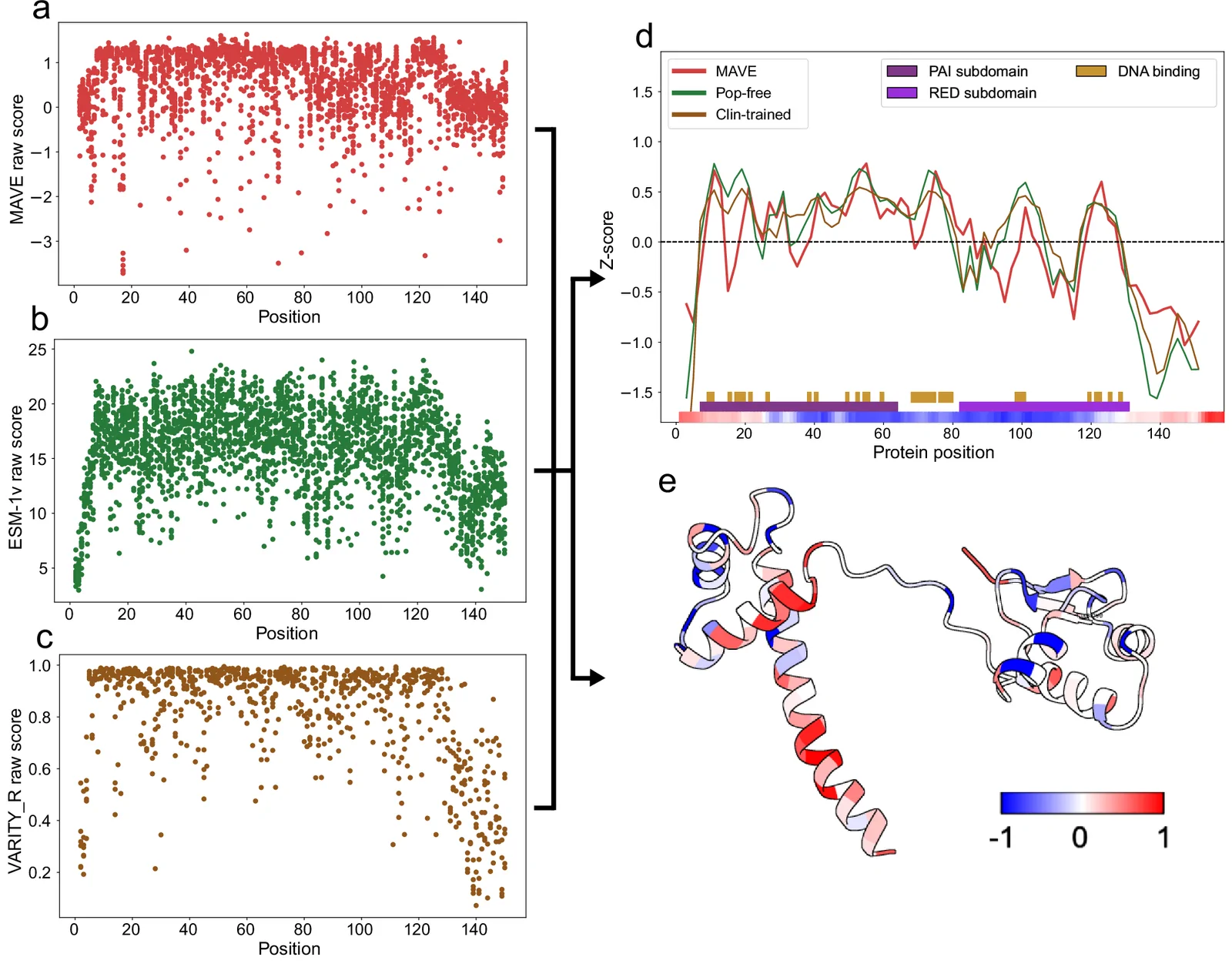
An illustration of MAVE and VEP dataset normalisation and comparison. MAVE data are shown in red, population-free VEP data in green, and clinical-trained VEP data in brown. **a** The average z-scores of the 5 population-free VEPs for all substitutions in the PAX6 protein. **b** The average z-scores of the 5 clinically trained VEPs for all possible SNVs in the PAX6 protein. **c** The MAVE dataset for the *PAX6* protein (le9 assay) converted into z-scores. **d** The three lines on the plot represent moving average z-scores for the population-free VEPs, clinically trained VEPs and the MAVE within a window of 5 residues and a stride of 2 residues. Higher values indicate more damaging predictions/measurements. Correspondence to notable protein features and a heatmap of AIUPred predictions are also shown on the plot (red indicates predicted disorder). **e** The difference between the MAVE z-score and the average population-free VEP z-score is mapped to the 3D structure of the PAX6 protein. Source data for the MAVE and VEP scores are provided in the Source Data file.

Figure 2 illustrates our approach. A comparison can be made between any two z-score converted MAVE or VEP score sets for a particular protein by subtracting one set of z-scores from the other, resulting in values close to 0 where the two sets agree perfectly and increasingly large positive or negative values in the case of extreme discordance. For the sake of consistency, all such z-score differences presented in this study represent the VEP being subtracted from the MAVE, resulting in positive scores for variants the MAVE finds more damaging, and negative scores for variants the VEP finds more damaging. Regions of discordance as measured by the z-score difference can then be associated with protein features, sites and domains, providing insight into the features of proteins that MAVEs and VEPs are most likely to disagree on. In Fig. 2, predictions from the population-free VEPs (Fig. 2a) and clinical-trained VEPs (Fig. 2b) are compared to a set of MAVE results for PAX6^4^ (Fig. 2c), plotted alongside notable protein features (Fig. 2d) and the difference scores mapped to the 3D protein structure (Fig. 2e). We repeated this across all 40 proteins.

To assess differences between MAVE and VEP score distributions, we compared the spread of z-scores across substitutions at the same residue. We quantified this difference by calculating the standard deviation of z-scores at each protein position, and averaging this per score-set for the 40 proteins for both population-free VEPs (Supplementary Fig. 2a) and clinical-trained VEPs (Supplementary Fig. 2b). The selected MAVE set has significantly higher positional standard deviation than all of the VEPs. This may reflect the fact that VEPs are constrained by generalised patterns learned from evolutionary and structural features, leading to more similar predictions across substitutions at a given position. By contrast, MAVEs measure variant effects empirically in a specific experimental context, allowing for a broader range of observed effects.

### Insight into amino acid-level differences

It is well known that mutations involving specific amino acids are much more likely to be damaging to protein function than others^44^. For example, aromatic amino acids are very bulky and hydrophobic, often driving the formation of the core of globular proteins. Mutations of these large residues to those with different properties, or the inclusion of these residues on the protein surface can lead to altered folding pathways or misfolding^45^. Cysteine residues are vital in the formation of disulphide bridges, particularly in extracellular proteins and those localised to oxidising compartments such as the endoplasmic reticulum. Additionally, the unique conformational constraints of proline and flexibility of glycine mean that substitutions at these positions often lead to significant changes in protein backbone structure.

We investigated the differences between MAVEs and VEPs in terms of their tendency to assign higher or lower impact scores to amino acid substitutions. To do this, we calculated the average difference in z-scores between MAVEs and VEPs for specific amino acid substitutions across the 40 proteins. Our results reveal systematic differences in how the two approaches evaluate particular classes of substitutions.

**Fig. 3:**
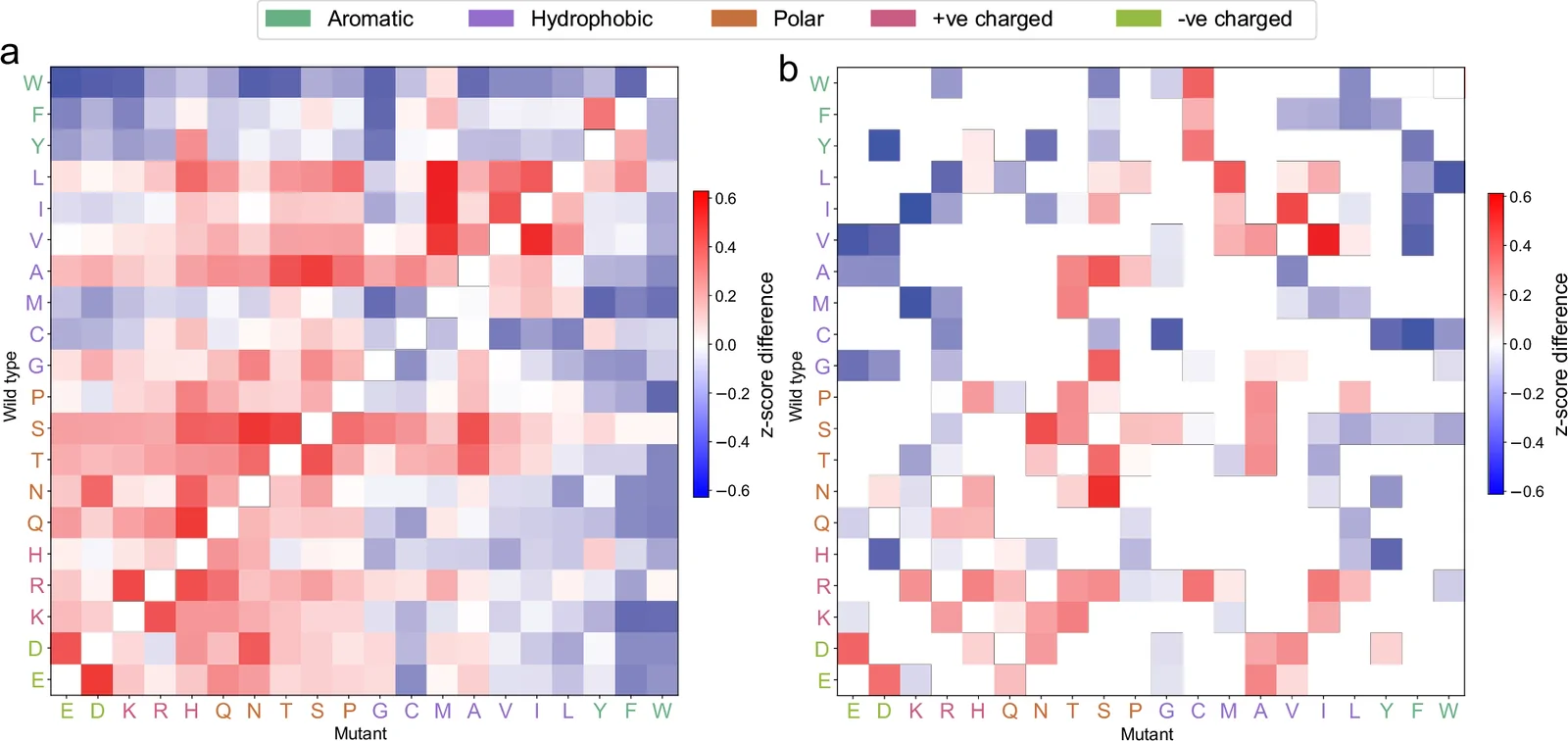
Heatmaps of the z-score differences between MAVEs and VEPs for each amino acid substitution. Positive, red cells indicate substitutions that MAVEs find more damaging than VEPs, while negative, blue cells indicate substitutions that VEPs find more damaging than MAVEs. White squares indicate substitutions where both methods are concordant. The general properties of the amino acids are indicated by the colour of the axis labels. **a** The difference between the MAVEs and population-free VEPs. **b** The difference between the MAVEs and clinically trained VEPs. Source data for the heatmaps are provided in the Source Data file.

Figure 3a shows the differences between the population-free VEPs and MAVEs, while Fig. 3b shows the same comparison for the clinically trained group of VEPs. Gaps in the latter heatmap arise because the clinically trained predictors can only score substitutions accessible via single nucleotide changes (~30% of possible amino acid substitutions). In both these heatmaps, positive (red) values indicate substitutions scored as more damaging by MAVEs, whereas negative (blue) values indicate those scored as more damaging by VEPs.

Several consistent trends are evident across both heatmaps. Mutations involving bulky hydrophobic residues (e.g., W, Y, F), either as the wild-type or mutant, are typically scored as more damaging by VEPs. A similar pattern is observed for cysteine residues. VEPs also tend to assign higher impact to substitutions involving large changes in physicochemical properties, such as charged to hydrophobic or hydrophobic to charged/polar transitions. In contrast, MAVEs tend to assign higher impact to substitutions between residues with similar properties (e.g., charged/polar to charged/polar or small hydrophobic to small hydrophobic).

We observed a notable difference between the population-free and clinically trained VEPs for substitutions from small hydrophobic residues (L, I, V, A) to charged or polar residues (E, D, K, R, Q, N). These substitutions are generally scored as more damaging than MAVEs by clinically trained VEPs, but less damaging by population-free VEPs. Direct comparison of VEP z-scores confirms this pattern, with clinical-trained predictors assigning significantly higher impact to nearly all such substitutions (Supplementary Data 4). Overall, population-free VEPs show greater concordance with MAVE data for this class of substitutions.

Mutations involving large physicochemical changes or residues with unique properties are generally considered more likely to be damaging, and these are consistently assigned higher impact by VEPs than by MAVEs. This pattern is consistent with VEPs relying on generalised evolutionary and structural constraints, in which similar substitutions are typically deleterious. These observations are also in line with previous studies showing that pathogenic variants are enriched in conserved, buried, and functionally important residues^46–49^. By contrast, MAVEs measure variant effects in a specific experimental context and therefore capture cases where these general trends do not hold. Conversely, substitutions between residues with similar properties, which are often assumed to be benign, are more frequently assigned higher impact by MAVEs, suggesting that the impact of mutations at these residues may be underestimated by VEPs.

To investigate whether the relationship between MAVEs and VEPs at specific amino acid substitutions is influenced by the MAVE methodology, we repeated the analysis by subsetting the MAVE dataset by assay type. Our findings (Supplementary Fig. 3) show a generally similar pattern of MAVE/VEP disagreement for each of the assay types, although the pattern becomes less clear for stability and trafficking assays, possibly due to the lower number of MAVE assays in these groups. This implies a degree of consistency across MAVE datasets despite differences in assay design, with broad patterns of agreement and disagreement with VEPs largely preserved. Differences between assays are therefore more likely to be evident at the level of specific protein features or individual variants.

### Protein features with discordant MAVE and VEP predictions

#### Variants at buried residues are predicted more damaging by VEPs

To examine where MAVE and VEP predictions diverge, we compared z-score differences between VEPs and MAVEs across structural residue classes, distinguishing residues that are surface-exposed, buried within the protein interior, or located at intermolecular interfaces (Supplementary Fig. 4). Surface residues show the highest positive z-score differences, whereas interface residues show the lowest, with interior residues intermediate, for both population-free and clinically trained VEPs.

**Fig. 4:**
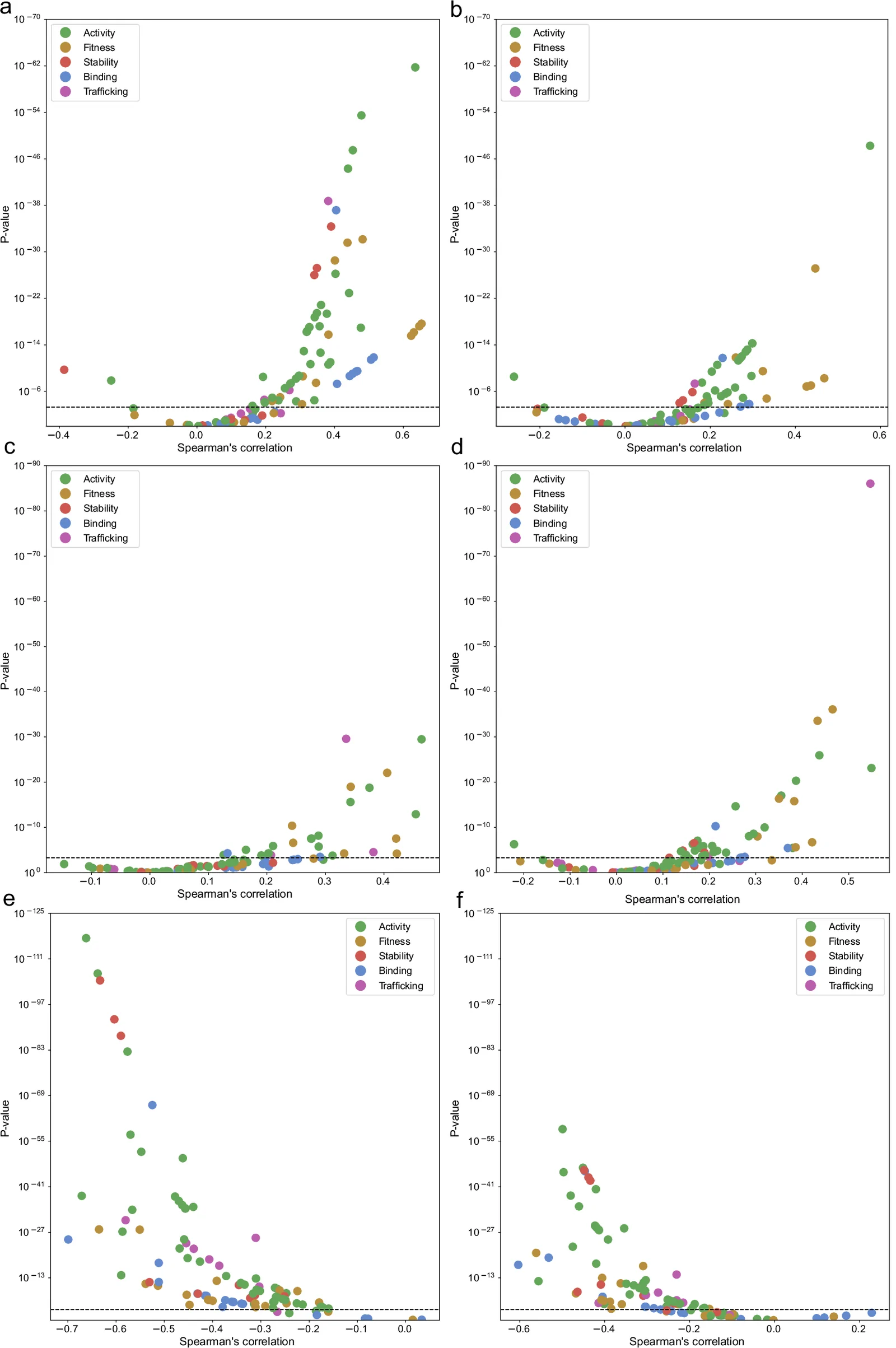
The correlation between z-score difference and protein properties. The Spearman’s correlation (two-sided) between average z-score difference at the residue level and protein properties. The y-axis indicates the significance of the correlation, and the dotted line represents the Bonferroni-corrected significance threshold. MAVE datasets are coloured by assay type. **a** The correlation between z-score difference and relative solvent accessibility (RSA) for population-free VEPs. **b** The correlation between z-score difference and RSA for clinically trained VEPs. **c** The correlation between z-score difference and AIUPred disorder predictions for population-free VEPs. **d** The correlation between z-score difference and AIUPred disorder predictions for clinically trained VEPs. **e** The correlation between z-score difference and residue conservation for population-free VEPs. **f** The correlation between z-score difference and residue conservation for clinically trained VEPs. Source data for these plots are provided in the Source Data file.

To further investigate this relationship, we calculated, for each MAVE dataset, the Spearman’s correlation between the mean z-score difference per position and the relative solvent accessibility (RSA) of the wild type at that position (Fig. 4a, b). Most datasets displayed a positive correlation between the z-score difference and the RSA (91/99 for population-free; 84/99 for clinical-trained), with 62 and 42 remaining significant after Bonferroni correction. Two datasets (MLH1 and PPARG) show a significant negative correlation.

Together, these analyses demonstrate a clear relationship between MAVE/VEP discordance and solvent accessibility. This is consistent with the substitution-level patterns observed in Fig. 3, where bulky hydrophobic residues, which are enriched in the protein interior and at interfaces, tend to show negative z-score differences. The strength of this relationship varies across datasets, ranging from weak negative to moderately strong positive correlations. These differences do not appear to be driven by assay type and may instead be protein-specific, as multiple MAVE datasets for the same protein tend to show similar correlation patterns in Fig. 4a and 4b.

#### Variants in intrinsically disordered regions are scored more damaging by MAVEs

We used AIUPred^50^ to predict intrinsic disorder for each protein. As with RSA, we calculated, for each MAVE dataset, the correlation between mean z-score difference and predicted disorder (Fig. 4c, d). Most datasets show a positive correlation between disorder and z-score difference (86/99 for population-free VEPs; 82/99 for clinical-trained VEPs), with 38 and 27 remaining significant after Bonferroni correction.

VEP performance is known to be reduced in intrinsically disordered regions^51,52^. These regions are typically less evolutionarily conserved than structured regions, and evolutionary conservation is a key feature in most VEPs (and in some cases the primary signal)^39,53^. Consistent with this, protein language model predictors such as ESM-1b and ESM-1v show broadly similar trends in intrinsically disordered regions to alignment-based methods such as GEMME, with no clear advantage in IDRs^51^. As a result, variants in disordered regions tend to be assigned lower impact by VEPs. Although intrinsically disordered regions are enriched in benign variation, both MAVEs and VEPs tend to assign predominantly low-impact scores to variants in these regions, with MAVEs showing a relative shift toward higher scores compared to VEPs. VEPs may therefore underestimate the impact of variants in these regions due to their reliance on conservation. By contrast, MAVEs are not constrained by evolutionary conservation and can capture context-specific effects in disordered regions. The observed pattern is therefore consistent with VEPs systematically assigning lower impact to variants in disordered regions relative to MAVEs.

#### Conserved residues are predicted more damaging by VEPs

We next assessed the relationship between evolutionary conservation and MAVE/VEP discordance. Sequence alignments were generated using MMSeqs2^54^, and conservation at each position was calculated as the fraction of sequences in the alignment that retain the wild-type amino acid (excluding gaps). We then calculated the correlation between mean per-position z-score difference and conservation (Fig. 4e-f).

Of all the protein attributes tested, conservation showed the strongest correlation with z-score difference, with some correlations approaching −0.7. Nearly all datasets displayed a negative correlation (97/99 for population-free VEPs; 94/99 for clinical-trained VEPs), with 90 and 69 remaining significant after Bonferroni correction. No dataset showed a significant positive correlation.

These results indicate that variants at highly conserved residues tend to be scored as more damaging by VEPs, whereas variants at less conserved positions tend to be scored as more damaging by MAVEs. This pattern is consistent with the strong reliance of VEPs on evolutionary conservation as a predictive feature. By contrast, MAVE scores are not directly constrained by conservation, and therefore may capture effects that are not reflected in evolutionary signals. Consistent with this, a simple substitution-based baseline using BLOSUM62 showed a broadly similar pattern of discordance with MAVE data (Supplementary Fig. 5a), supporting the conclusion that evolutionary conservation is a major contributor to these differences. Likewise, EVE^55^, a per-protein model that learns constraints solely from sequence alignments, shows a similar pattern of discordance with MAVE data (Supplementary Fig. 5b), indicating that these trends are not dependent on cross-protein generalisation but instead reflect shared reliance on evolutionary information.

### Characteristics of discordantly predicted protein regions

**Fig. 5:**
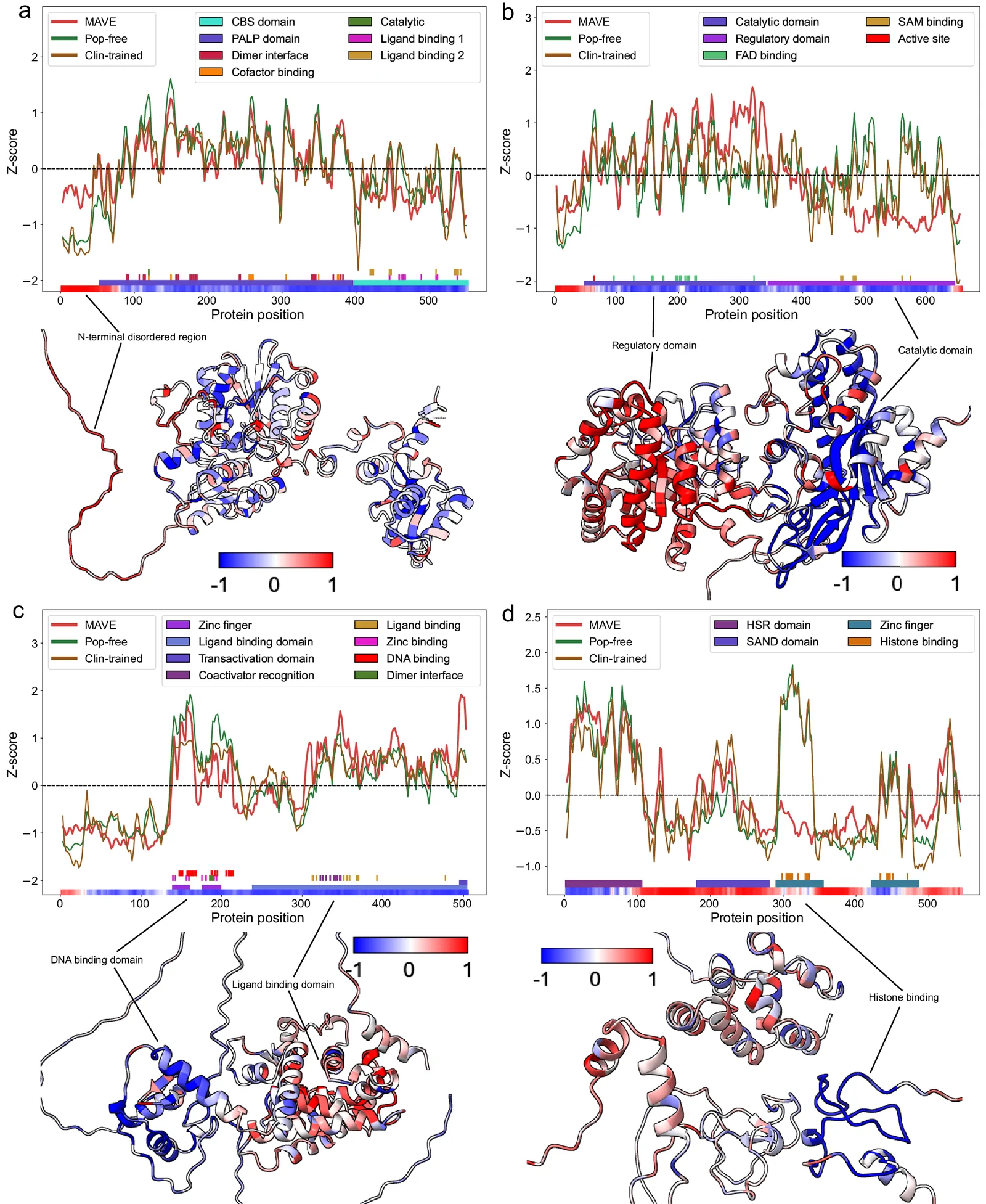
Correspondence between z-score difference and protein features. Moving averages of the MAVE z-scores, population-free VEP z-scores and clinically trained VEP z-scores within a sliding window of 5 residues are plotted against protein position. Notable protein features and a heatmap representing AIUPred disorder predictions (red indicates disorder, blue indicates order) are shown at the bottom of each chart. The difference between the MAVE and population-free VEP z-scores is mapped onto the AlphaFold-predicted protein structure. **a** CBS **b** MTHFR **c** PPARG **d** PRKN. Source data for these plots are provided in the Source Data file.

To identify protein regions associated with high discordance between MAVEs and VEPs, we analysed sequence- and residue-level annotations from multiple databases. For variants within each annotated region, we calculated the median z-score difference and assessed significance using the Wilcoxon signed-rank test. Selected examples are shown in Fig. 5, and the full set of annotations is provided in Supplementary Data 5.

Regions with the most significant positive z-score differences (MAVEs scoring variants as more damaging) are dominated by disorder annotations. In total, 11 of 40 proteins contain disordered regions with significantly positive differences. An example is CBS, where the N-terminal disordered region shows consistently higher z-scores in MAVEs than in VEPs (Fig. 5a). Although both methods generally assign low impact to variants in this region, VEPs systematically assign lower scores. This pattern is consistent with the broader trends observed above for predicted disordered regions.

Several structured domains also show significant positive differences, affecting 18 proteins. The strongest example is the regulatory domain of MTHFR, which shows consistent positive differences across all MAVE conditions (Fig. 5b), despite having a well-defined structure and minimal overlap with disordered regions. Other protein domains that demonstrate significant positive z-score differences include the C-terminal ligand-binding domain of PPARG (Fig. 5c), the Laminin domains of F9 (Supplementary Fig. 6a), the MSH2 domain between residues 300 and 516 (Supplementary Fig. 6b), and the C-terminal C2 domain of PTEN (Supplementary Fig. 6c).

Domain annotations are even more frequently associated with negative z-score differences (VEPs scoring variants as more damaging), affecting 27 proteins. In many cases, individual proteins contain both positively and negatively shifted domains. For example, in PPARG (Fig. 5c), the DNA-binding domain shows strongly negative differences, whereas the ligand-binding domain shows positive differences. Interestingly, the three domains with the lowest negative difference scores are all DNA-binding domains (in PPARG, CRX and AIRE), suggesting a consistent bias in how these regions are scored.

Binding sites are also enriched among regions with negative differences. For example, the most significant discordances are in a histone H3 binding site in AIRE (Fig. 5d), a ubiquitin binding site in PRKN, and a ligand binding site in HMBS. Transmembrane regions in KCNH2 and KCNE1 similarly show negative differences, consistent with patterns observed for hydrophobic and buried residues.

Active site annotations also show substantial discordance in several proteins, including PRKN, F9, and ASPA. The PRKN MAVE probed protein stability^25^, so the discordance is likely due to active site variants impacting protein function, but not stability. The situation is similar in F9, as the MAVE investigates the impact of variants on correct protein trafficking, rather than protein function and thus variants that disrupt protein activity may not impact trafficking^56^. For ASPA, both a stability and fitness assay^10^ are highly discordant with the VEPs across the 11 annotated active site residues. The fitness assay was carried out following the observation that some variants produced a toxic effect with no known underlying mechanism. Given the mechanism of toxicity remains unknown, we speculate that it is likely not due to changes in the native activity of the protein as the negative z-score difference in this region strongly implies that active site variants have no impact on the toxic effect.

We also investigated discordance between population-free and clinically trained VEPs. For each annotated region, we calculated the difference between the median z-scores of the two groups (Supplementary Data 6), where positive values indicate higher impact scores from population-free VEPs. As with the MAVE comparison, the largest differences occur in disordered regions. However, unlike the MAVE-VEP comparison, the direction of these differences is not consistent, with both positive and negative values observed. The magnitude of the differences is generally smaller than in the MAVE comparison, with notable exceptions including extended disordered regions in MLH1 and VHL. The largest positive differences are primarily observed at binding sites, including zinc-binding residues in TP53, ATP-binding residues in MET, and DNA-contact residues in CRX. Overall, differences between the two VEP groups are substantially smaller than those observed between MAVEs and VEPs, consistent with the generally higher concordance among VEP predictions.

### Analysis of discordantly predicted clinical variants

While the analyses above highlight systematic differences between MAVEs and VEPs, we next examine how these differences appear in clinically annotated variants. We analysed ClinVar variants annotated as pathogenic/likely pathogenic (2578) and benign/likely benign (1059) across the 40 proteins, calculating the z-score difference for each variant across all MAVEs and both population-free and clinically trained VEPs. The full distribution is shown in Supplementary Fig. 7a for population-free predictors and Supplementary Fig. 7b for clinically trained predictors and provided in Supplementary Data 7. In total, 684 pathogenic and 322 benign variants were discordant between a MAVE and VEP, defined as having a z-score difference exceeding one for either VEP class. We then examined five cases of substantial discordance to explore potential explanations.

#### BRCA1

Breast cancer type 1 susceptibility protein performs a central role in the homology-directed repair of DNA double-strand breaks^57^. BRCA1 also participates in non-homologous end-joining DNA repair^58^ and performs several other functions relevant to maintaining genome stability; it is classed as a tumour suppressor. Pathogenic variants in BRCA1 increase the risk of breast and/or ovarian cancer, typically through loss of protein function. Our dataset contains four MAVE score sets for BRCA1, produced by three independent groups, which each use different methodologies. One of the BRCA1 MAVEs used a saturation genome editing approach to introduce variants into the protein’s N-terminal RING domain and C-terminal BRCT domains in the HAP1 human cell line^9^. BRCA1 is an essential gene in HAP1 cells, and loss of function impairs cellular growth, permitting a readout of protein function by measuring growth rate.

Three variants in BRCA1 stand out as having both extremely high positive MAVE z-scores and negative VEP z-scores; A1823S, D1818G, and A1823T. These variants are all pathogenic, but are only identified as damaging by the SGE-based fitness assay, and not by the VEPs or the non-SGE activity-based MAVEs. The codon encoding A1823 straddles two exons, so the variants at that location may impact splicing. Pangolin^59^ predicts a 65% chance of splice donor loss on a threonine mutation (NM_007294.4(BRCA1):c.5467 G > A (p.Ala1823Thr)) and a 67% chance of splice loss on a serine mutation (NM_007294.4(BRCA1):c.5467 G > T (p.Ala1823Ser)). A study also found that D1818G impacts splicing and results in skipping of exon 22^60^. Nine of the ten VEPs in the analysis generate negative z-scores for these variants; the exception is SNPred, which produces positive z-scores for the two variants at A1823. It is likely the other VEPs are failing to account for the impact on splice sites, resulting in incorrect benign predictions for these variants. Because this MAVE was performed using saturation genome editing in a human cell line, the protein was able to be studied in its endogenous context, with native splicing machinery. This is likely what allowed for detection of these variants in the SGE-based MAVE and not in the activity-based MAVEs^61^, which were performed in human (HeLa) cells but utilised cDNA and thus were not sensitive to the impact of splice variants. The reason for SNPred identifying the likely splice variants is unclear, as it was not trained with any splicing data, although it was evaluated by its authors using the SGE BRCA1 dataset^31^.

#### SNCA

α-synuclein is a protein that plays an important role in synaptic activity. Variants in α-synuclein can cause Parkinson’s disease and other neurodegenerative conditions collectively called α-synucleinopathies^62^. Pathogenic variants typically trigger aberrant aggregation of α-synuclein into structures such as Lewy bodies that impair normal cellular functions. The MAVE for α-synuclein activity was performed in yeast cells at different synuclein concentrations, with the readout being the yeast growth rate, which is impaired by, and thus a proxy for, α-synuclein aggregation^63^.

The A53T variant in SNCA received a negative z-score from all of the population-free VEPs, while the MAVE and clinical-trained VEPs produced z-scores closer to zero, implying a lack of confidence in the prediction. A53T is well known to have a high aggregation potential and is implicated in Parkinson’s disease^64^. Using an alignment of 1051 sequences related to α-synuclein generated by MMSeqs2^54^, we found that threonine is actually the most common residue at the equivalent position to alanine 53 in the human sequence (401 occurrences vs only 84 for alanine) (Supplementary Fig. 8a). The most likely explanation is that population-free VEPs are failing to correctly predict this mutation because the human pathogenic mutant residue is common at this position through evolution. Given that this is a well-known pathogenic mutation with extensive characterisation, the population-free VEPs are unquestionably incorrect in their non-damaging assessment, likely because this mutation violates their baseline assumption that evolutionary conservation is a good proxy for variant effect. The exact reason why this assumption does not hold in this case is unclear, but it may be the case that the pathogenic effect of this variant only becomes evident at a post-reproductive age and is thus not strongly selected against. The clinical-trained VEPs and MAVE fared better, but were still not entirely concordant with the ClinVar classification, implying that the variant had some effect on protein function but no certainty whether that effect would translate into a phenotype.

#### MSH2

The primary role of DNA mismatch repair protein MSH2 is to locate and repair DNA mismatches, which is a function it performs as a heterodimer with MSH6, forming the MutSα complex. Alternatively, MSH2 can dimerise with MSH3, forming the MutSβ complex, which repairs larger-scale insertions and deletions^65^. Pathogenic variants in MSH2 cause Lynch syndrome, an autosomal dominant disease that greatly increases cancer susceptibility. Many of the variants that cause Lynch syndrome result in decreased mismatch repair activity^66^. The MAVE for MSH2 was carried out in human HAP1 cells, with endogenous expression of MSH2 knocked out; mutant MSH2 was introduced using a lentiviral construct^67^. Selection was performed using a purine analogue that is toxic to cells with a functional mismatch repair pathway, resulting in higher growth rates for cells with reduced MSH2 activity (i.e., a reverse-survival assay, where cells with impaired MSH2 activity are better able to proliferate than the wild type).

The S612P MSH2 variant is classified as “Likely benign” in ClinVar and receives a high positive z-score from all VEPs but a low negative z-score from the MAVE, indicating substantial discordance. This variant occurs in an alpha helix, which proline mutations tend to disrupt, and in an alignment of 13624 sequences by MMSeqs2, only five occurrences of proline were observed at this position, demonstrating strong residue constraint (Supplementary Fig. 8b). Furthermore, no variant in S612 is present in gnomAD and numerous other pathogenic variants occur in the same helix (A604D, D603Y/G/V, A600D/V). These observations tend to concur with the VEP interpretation of likely pathogenicity rather than the MAVE and ClinVar label. A closer investigation of the ClinVar evidence supporting a classification of “likely benign” reveals that the MSH2 MAVE study is actually used as functional evidence for the assignment^68^, potentially explaining the correspondence between the ClinVar classification and the MAVE.

#### MTHFR

Methylenetetrahydrofolate reductase is a metabolic enzyme that catalyses the conversion of 5,10-methylenetetrahydofolate to 5-methyltetrahydrofolate, which serves as a methyl group donor for the conversion of homocysteine into methionine^69^. Variants that cause a severe loss of MTHFR function (usually defined as <20% of wild-type function remaining) cause homocystinuria, an autosomal recessive disease that results in developmental delay and severe intellectual disability^70^. The MAVE for MTHFR makes use of the ability of the human MTHFR protein to functionally complement its yeast homologue *MET13*, which is essential for yeast growth unless supplemental methionine is added to the growth medium^71^. Variant function was measured by assessing the yeast growth rate via a Tile-seq approach in the presence of different concentrations of folinate.

The MTHFR MAVE sets all have numerous pathogenic variants with positive z-scores from all VEPs and negative MAVE z-scores. Two such variants are Y506D and P572L, which are classed as “Likely pathogenic” and “Pathogenic/likely pathogenic” variants in ClinVar, respectively. Y506 is a core residue at a dimerisation interface. This particular variant was not remarked on by the authors of the MAVE study but it was noted that dimerisation interface residues scored no lower than other residues at the surface of the same domain^71^. This was attributed to dimerisation not being strictly required for MTHFR activity, although the pathogenic classification of the variant implies that some potentially unaccounted-for function of the protein is impacted by mutation of the interface. One patient study found that a compound heterozygote bearing the Y506D variant along with a partial duplication in the other allele (p.Leu89_Pro101dup) had only 8.1% MTHFR activity remaining^70^. P572 is a fully buried residue; mutation to leucine is reported as pathogenic and likely pathogenic in ClinVar by two submitters, and additionally has been found to severely reduce MTHFR activity in a human expression system (3.1 ± 0.4% activity remaining)^72^. It is likely, in both cases, that the VEPs successfully identified damaging variants that were overlooked by the MAVE, possibly due to experimental noise, or a difference between the yeast expression system and human gene context.

#### CARD11

Caspase recruitment domain-containing protein 11 is a signalling protein involved in the adaptive immune response following the activation of T and B cell surface receptors. It is activated by phosphorylation, homooligomerises and recruits BCL10 and MALT1, leading to the phosphorylation of downstream proteins and eventual release of NF-κB^73^. CARD11 variants can cause B-cell expansion with NFKB and T-cell anergy, an autosomal dominant condition characterised by splenomegaly (enlarged spleen) and polyclonal B-cell expansion^74^. CARD11 variants can also cause both dominant and recessive forms of immunodeficiency^75,76^. The MAVEs for CARD11 activity used a saturation genome editing approach in a human B-cell lymphoma cell line, TMD8, where growth is dependent on NF-κB signalling and therefore upstream CARD11 and B-cell receptor signalling as well^77^. As CARD11 activity is vital for growth in this cell line, relative fitness was determined by the abundance of each variant following selection. A second assay was performed by the same group using an upstream signalling inhibitor to identify potential gain-of-function variants.

C49Y is a pathogenic variant that is assigned very low negative z-scores by both MAVE assays in CARD11, but positive z-scores by all the VEPs. C49 is a hotspot for gain-of-function variants in CARD11, where multiple variants increase downstream NF-κB activity^77^. The difference between MAVE and VEP predictions at this location is caused by a disconnect in readouts between the two approaches. VEPs are accurately predicting the variant as damaging (in terms of human disease), while the MAVEs are accurately capturing the increased downstream activity caused by the variant – however this fitter-than-wild type variant looks non-damaging in a direct comparison of the scores due to the z-score normalisation. In this particular fitness assay, gain-of-function variants do not negatively impact cellular fitness as they would in the context of the whole organism. This is a limitation of this analysis rather than a meaningful difference between the MAVE and VEP scores, as both are flagging the variant as functionally abnormal. The same caveat applies to other gain-of-function variants.

## Discussion

Understanding both the strengths and limitations of variant interpretation methods is essential for improving clinical classification of variants of uncertain significance. Our analysis highlights key systematic differences between MAVEs and VEPs, two of the key techniques that provide evidence for variant effects. VEPs are strongly influenced by the signal of evolutionary conservation, which leads to them making more damaging predictions than MAVEs in certain regions, including buried residues, interfaces and conserved residues. The opposite trend occurs on the protein surface and within disordered regions. MAVEs, in contrast, can provide direct functional evidence but are limited by experimental noise and the choice of assay, which may not capture all disease-relevant mechanisms.

Expanding the use of MAVEs to assay distinct phenotypes is critical. A single assay may miss important variant effects. For example, VAMP-seq may overlook catalytic impairment at active sites. Applying different assay modalities allows protein functions to be dissected more precisely, offering insights into gain-of-function, dominant-negative, or splicing mechanisms that would otherwise go undetected. Saturation genome editing (SGE) represents a major step forward in this area, enabling variant assessment within native genomic contexts and avoiding artefacts introduced by overexpression systems.

With the continued improvement of computational variant effect predictor technology, correct prediction of cases similar to those highlighted here where the VEPs failed to correctly classify ClinVar-labelled variants will become more important. A remarkable amount of progress has been made by focussing purely on evolutionary conservation, and VEPs that solely make use of this feature remain among the top performers to date (although in the case of SNCA, the over-reliance on conservation also proved a hindrance). Several recent predictors have benefitted from the addition of protein structural features on top of conservation^78^, implying that the path to further progress may be from integration of alternative sources of informative features. One such feature that may lead to improvements is the addition of a splice-site impact predictor, which may have allowed more of the VEPs to correctly identify the three splice-impacting variants in BRCA1 as pathogenic.

From the analysis of amino acid substitutions and the performance of VEPs in both disordered regions and at conserved residues, it does appear that VEP scores tend to be influenced by protein features to a greater extent than MAVEs. Many of the quirks of VEP predictions we identified are rooted in their reliance on evolutionary conservation, either directly or indirectly, leading to strong performance in most regions at the expense of poor performance in unusual or poorly characterised regions that do not occur often enough or contain enough clinically relevant variants to significantly reduce overall performance metrics. Further evidence of this is seen in the VEPs’ inability to identify disease mechanisms that do not fit the standard loss-of-function paradigm among the most discordantly predicted variants and their general tendency to perform worse at gain-of-function and dominant-negative variants^79^. While MAVE scores are less likely to be influenced by the local protein environment, limitations in assay design can lead to variant misinterpretation and discordance with other sources of evidence.

There was no substantial link between the methodology underlying a MAVE and its discordance with VEP predictions on the scale of full protein sequences. In Fig. 4, MAVEs from all 5 categories demonstrated significant correlations with protein attributes. Some differences were observed for annotated regions in proteins where multiple types of assays were performed. For example, 11 residues annotated as a phosphopeptide binding site in CHEK2 had a significant negative difference score from a fitness assay, but a significant positive difference score from an activity assay. Another example is the “non-cytoplasmic domain” annotation in KCNE1, which had significant positive z-score differences in the trafficking assay, but not for the activity assay. Differences in discordance driven by MAVE methodology are most evident at the level of specific mutations, such as those seen at important functional residues in proteins assessed with VAMP-seq or other assays not focussed on assessing protein function.

A related question, which we have not explored in detail here, is the extent to which different VEPs agree with one another. While our analysis focuses on the relationship between VEPs and MAVEs, inter-predictor variability is substantial and may itself be informative. Recently, we systematically examined concordance across multiple VEPs at proteome scale and found that agreement varies widely across proteins, reflecting differences in model features and training paradigms rather than intrinsic predictability^41^. Notably, inter-VEP agreement showed little relationship with agreement to experimental data, suggesting that predictor consensus should not be interpreted as a proxy for accuracy. Instead, regions of disagreement may highlight mechanistic blind spots and provide a rationale for prioritising experimental follow-up.

While the generation of MAVE datasets for all human proteins and isoforms is an aspirational goal of the Atlas of Variant Effects Alliance (AVE), it is, for the moment, not yet attainable^80^. Although mutational scanning is becoming increasingly widespread, generating high-quality datasets remains resource-intensive and often requires assay designs tailored to specific protein functions. Consequently, VEPs continue to play a key role by providing rapid, scalable predictions across the many proteins and variants that are unlikely to be assayed experimentally in the near term.

In conclusion, while modern VEPs and MAVEs show a strong correlation and tend to be in concordance for most clinically relevant variants, the cases where major discordance emerges shed light on the weaknesses of both methods. Understanding these differences provides a basis for improving both methods and for more accurately capturing the diverse mechanisms underlying variant effects.

## Methods

### Data sources

Deep mutational scan datasets were obtained from several sources, including their primary publication (Supplementary Data 1), MaveDB^81^ and ProteinGym^19^.

For the variant effect predictors, pre-computed scores were obtained for CPT-1 from the CPT Zenodo repository (https://zenodo.org/records/8140323). ESCOTT predictions were obtained from the PRESCOTT Zenodo repository (https://zenodo.org/records/10670914). GEMME predictions were calculated locally using alignments generated by MMseqs2 and the GEMME Docker container. SaProt was run locally using the instructions in the GitHub repository (https://github.com/westlake-repl/SaProt). ESM-1v was run locally using the ‘zero shot variant effect prediction’ notebook as a template (https://github.com/facebookresearch/esm/tree/main/examples/variant-prediction). VARITY predictions were obtained from the VARITY webserver (http://varity.varianteffect.org/). mvPPT predictions were obtained from the mvPPT website (http://www.mvppt.club/). SNPred predictions were downloaded from Synapse (https://www.synapse.org/Synapse:syn52137034/files/). MetaRNN, MutFormer, REVEL and BayesDel predictions were obtained from dbNSFP version 5.1^32^. EVE and popEVE predictions were obtained in bulk from their respective websites (https://evemodel.org/ and https://pop.evemodel.org/).

Pathogenic and benign variants were labelled using ClinVar (16/10/2025 release). We used ClinVar pathogenic variants with a 1-star or higher review status as the pathogenic dataset. Putatively benign variants were labelled using gnomAD version 4.1. No additional filtering or sub-setting of gnomAD variants was applied beyond using the gnomAD internal quality filter. While gnomAD is not a purely benign set of variants, we refer to it as ‘putatively benign’ as it provides a snapshot of genetic variation in a healthy human population. While gnomAD is likely to contain some recessive and low-penetrance pathogenic variants, particularly among variants with low allele frequency, it better reflects the real-life clinical application of MAVEs and VEPs than other purely benign alternatives, namely distinguishing rare pathogenic variants from rare benign variants. Thus, we have not filtered the dataset for allele frequency or other attributes.

Disorder predictions were obtained from the AIUPred web tool (https://aiupred.elte.hu/).

Protein structures used to calculate surface, interior and interface annotations were obtained from the Protein Data Bank (https://www.rcsb.org/). Predicted structures were obtained from AlphaFold (https://alphafold.ebi.ac.uk/).

We used the InterPro API^82^ to obtain sequence-level annotations from several databases including FunFam^83^, MobiDB^84^, ELM^85^, Phobius^86^ and Coils^87^, as well as residue-level annotations from PIRSF^88^ and CDD^89^.

### MAVE assay group selection

Results obtained from analyses where multiple MAVE datasets are pooled, such as those shown in Fig. 3, S1, S2, S3, S4, and S5 have the potential to be skewed by proteins with multiple available MAVEs. To address this, we selected only a single MAVE dataset per-protein on the basis of the greatest AUROC to represent the MAVE in these analyses. The 40 selected MAVE datasets are indicated in Supplementary Data 1.

### AUROC calculation

Area under the receiver operating characteristic curve (AUROC) was calculated using the roc_auc_score() function of the sklearn Python package (0.18.1). Prior to AUROC calculation, any dataset with an inverted scale was subtracted from 1 to ensure that a score of 0 represented wild-type-like variants and 1 represented the greatest fitness impact.

### Statistical analysis

Statistical analyses were performed using the SciPy Python package (1.15.2). The functions used include scipy.stats.spearmanr() for the calculation of Spearman’s ρ, scipy.stats.wilcoxon() for Wilcoxon’s signed-rank test and scipy.stats.mannwhitneyu() for the Mann-Whitney U-test.

### Structural properties

We calculated the relative solvent accessibility (RSA) using both biological assemblies from the PDB and AlphaFold structures (Supplementary Data 8 and 9). RSA was calculated for each residue using the Shrake-Rupley algorithm^90^.

The classifications of surface, interior and interface were assigned by RSA^91^. RSA was calculated from the first biological assembly of each protein in the PDB. Interfaces are defined as areas where the RSA changes between the monomeric and complex assembly structures. Surface residues have an RSA of 25% or higher, while interior residues have an RSA less than 25%. Among interface residues, support residues have an RSA of less than 25% in both the monomer and complex. Interface rim residues have an RSA of 25% or higher in both the monomer and complex. Core interface residues have an RSA of 25% or higher in the monomer but less than 25% in the complex. We previously found that interface rim residues were very similar to surface residues in terms of properties and pathogenic variant enrichment, so all calculations using these definitions include the interface rim as surface residues^49^. See Supplementary Data 9 for the full classification of PDB residues used.

While PDB-derived structures were useful for defining interface residues with proteins and other biomolecules, the greater sequence coverage of AlphaFold structures, particularly within disordered regions, made them more useful for subsequent analyses. As such, Fig. 4A was calculated with RSA derived from AlphaFold structures and Fig. 5 and S6 also display AlphaFold structures.

### Reporting summary

Further information on research design is available in the Nature Portfolio Reporting Summary linked to this article.

## Supplementary information

Supplementary Information

Description of Additional Supplementary Files

Supplementary Data 1

Supplementary Data 2

Supplementary Data 3

Supplementary Data 4

Supplementary Data 5

Supplementary Data 6

Supplementary Data 7

Supplementary Data 8

Supplementary Data 9

Reporting Summary

Transparent Peer Review file

## Source data

Source Data

## Data Availability

The MAVE and VEP variant effect scores analysed in this study are available from Figshare (https://doi.org/10.6084/m9.figshare.29828504). The datasets generated during this study are provided as Supplementary Data 1–9. AlphaFold and PDB data sources used are referenced in Supplementary Data 8. Source data are provided with this paper. All other data supporting the findings of this study are available within the article and its Supplementary Information files. Source data are provided with this paper.
