# Supplementary material for: Why variant effect predictors and multiplexed assays agree and disagree
Source: Nat Commun. 2026 Aug 27;17:10295. doi: 10.1038/s41467-026-77211-x (PMC13620131; doi:10.1038/s41467-026-77211-x)
Supplement: Supplementary file 1 [file 41467_2026_77211_MOESM12_ESM.pdf]

nature portfolio

Corresponding author(s): Joseph Marsh, Benjamin Livesey

Last updated by author(s): 2026/04/28

Reporting Summary

Nature Portfolio wishes to improve the reproducibility of the work that we publish. This form provides structure for consistency and transparency in reporting. For further information on Nature Portfolio policies, see our Editorial Policies and the Editorial Policy Checklist.

Statistics

For all statistical analyses, confirm that the following items are present in the figure legend, table legend, main text, or Methods section.

n/a	Confirmed	
☐	☒	The exact sample size (*n*) for each experimental group/condition, given as a discrete number and unit of measurement
☒	☐	A statement on whether measurements were taken from distinct samples or whether the same sample was measured repeatedly
☐	☒	The statistical test(s) used AND whether they are one- or two-sided<br>*Only common tests should be described solely by name; describe more complex techniques in the Methods section.*
☐	☒	A description of all covariates tested
☐	☒	A description of any assumptions or corrections, such as tests of normality and adjustment for multiple comparisons
☐	☒	A full description of the statistical parameters including central tendency (e.g. means) or other basic estimates (e.g. regression coefficient) AND variation (e.g. standard deviation) or associated estimates of uncertainty (e.g. confidence intervals)
☐	☒	For null hypothesis testing, the test statistic (e.g. *F*, *t*, *r*) with confidence intervals, effect sizes, degrees of freedom and *P* value noted<br>*Give P values as exact values whenever suitable.*
☒	☐	For Bayesian analysis, information on the choice of priors and Markov chain Monte Carlo settings
☒	☐	For hierarchical and complex designs, identification of the appropriate level for tests and full reporting of outcomes
☐	☒	Estimates of effect sizes (e.g. Cohen's *d*, Pearson's *r*), indicating how they were calculated

Our web collection on statistics for biologists contains articles on many of the points above.

Software and code

Policy information about availability of computer code

Data collection

For the variant effect predictors, pre-computed scores were obtained for CPT-1 from the CPT zenodo repository (https://zenodo.org/records/8140323). ESCOTT predictions were obtained from the PRESCOTT zenodo repository (https://zenodo.org/records/10670914). GEMME predictions calculated locally using alignments generated by MMseqs2 and the GEMME docker container. SaProt was run locally using the instructions in the github repository (https://github.com/westlake-repl/SaProt). ESM-1v was run locally using the 'zero shot variant effect prediction' notebook as a template (https://github.com/facebookresearch/esm/tree/main/examples/variant-prediction). VARITY predictions were obtained from the VARITY webserver (http://varity.varianteffect.org/). mvPPT predictions were obtained from the mvPPT website (http://www.mvppt.club/). SNPred predictions were downloaded from Synapse (https://www.synapse.org/Synapse:syn52137034/files/). MetaRNN, MutFormer, REVEL and BayesDel predictions were obtained from dbNSFP version 5.132. EVE and popEVE predictions were obtained in bulk from their respective websites (https://evemodel.org/ and https://pop.evemodel.org/).

Data analysis

Area under the receiver operating characteristic curve (AUROC) was calculated using the roc_auc_score() function of the sklearn python package (0.18.1). Prior to AUROC calculation, any dataset with an inverted scale was subtracted from 1, to ensure that a score of 0 represented wild type-like variants and 1 represented the greatest fitness impact.

Statistical analyses were performed using the scipy python package (1.15.2). The functions used include scipy.stats.spearmanr() for the calculation of Spearman's ρ, scipy.stats.wilcoxon() for Wilcoxon's signed rank test and scipy.stats.mannwhitneyu() for the Mann Whitney U-test.

For manuscripts utilizing custom algorithms or software that are central to the research but not yet described in published literature, software must be made available to editors and reviewers. We strongly encourage code deposition in a community repository (e.g. GitHub). See the Nature Portfolio guidelines for submitting code & software for further information.

Data

Policy information about availability of data

All manuscripts must include a data availability statement. This statement should provide the following information, where applicable:

- Accession codes, unique identifiers, or web links for publicly available datasets
- A description of any restrictions on data availability
- For clinical datasets or third party data, please ensure that the statement adheres to our policy

The raw MAVE and VEP datasets used in this study are available from Figshare, doi: 10.6084/m9.figshare.29828504

Research involving human participants, their data, or biological material

Policy information about studies with human participants or human data. See also policy information about sex, gender (identity/presentation), and sexual orientation and race, ethnicity and racism.

Reporting on sex and gender	Sax and gender were not relevant to this study
Reporting on race, ethnicity, or other socially relevant groupings	Race and ethnicity were not relevant to this study
Population characteristics	Population characteristics were not relevant to this study
Recruitment	No recruitment took place.
Ethics oversight	No ethics oversight was necessary.

Note that full information on the approval of the study protocol must also be provided in the manuscript.

Field-specific reporting

Please select the one below that is the best fit for your research. If you are not sure, read the appropriate sections before making your selection.

☒ Life sciences ☐ Behavioural & social sciences ☐ Ecological, evolutionary & environmental sciences

For a reference copy of the document with all sections, see nature.com/documents/nr-reporting-summary-flat.pdf

Life sciences study design

All studies must disclose on these points even when the disclosure is negative.

Sample size	All calculations were performed with as much data as possible.
Data exclusions	MAVE datasets were excluded from this study on the basis of variant availability (1000 variant minimum), study quality (defined methodology) and a link to human disease (5 pathogenic variants in ClinVar). MAVE datasets from large stability-based studies were also excluded as they could have skewed the analyses.
Replication	The analyses were performed across 99 MAVE datasets and 10 top-performing VEPs to ensure reproducibility of the trends observed.
Randomization	No randomization was performed or necessary.
Blinding	No blinding was performed or necessary

Reporting for specific materials, systems and methods

We require information from authors about some types of materials, experimental systems and methods used in many studies. Here, indicate whether each material, system or method listed is relevant to your study. If you are not sure if a list item applies to your research, read the appropriate section before selecting a response.

Materials & experimental systems

n/a	Involved in the study
☒	☐ Antibodies
☒	☐ Eukaryotic cell lines
☒	☐ Palaeontology and archaeology
☒	☐ Animals and other organisms
☒	☐ Clinical data
☒	☐ Dual use research of concern
☒	☐ Plants

Methods

n/a	Involved in the study
☒	☐ ChIP-seq
☒	☐ Flow cytometry
☒	☐ MRI-based neuroimaging

Plants

Seed stocks	*Report on the source of all seed stocks or other plant material used. If applicable, state the seed stock centre and catalogue number. If plant specimens were collected from the field, describe the collection location, date and sampling procedures.*
Novel plant genotypes	*Describe the methods by which all novel plant genotypes were produced. This includes those generated by transgenic approaches, gene editing, chemical/radiation-based mutagenesis and hybridization. For transgenic lines, describe the transformation method, the number of independent lines analyzed and the generation upon which experiments were performed. For gene-edited lines, describe the editor used, the endogenous sequence targeted for editing, the targeting guide RNA sequence (if applicable) and how the editor was applied.*
Authentication	*Describe any authentication procedures for each seed stock used or novel genotype generated. Describe any experiments used to assess the effect of a mutation and, where applicable, how potential secondary effects (e.g. second site T-DNA insertions, mosiacism, off-target gene editing) were examined.*
