# Supplementary material for: Why variant effect predictors and multiplexed assays agree and disagree
Source: Nat Commun. 2026 Aug 27;17:10295. doi: 10.1038/s41467-026-77211-x (PMC13620131; doi:10.1038/s41467-026-77211-x)
Supplement: Supplementary file 2 [file 41467_2026_77211_MOESM13_ESM.pdf]

nature portfolio

Peer Review File

Why variant effect predictors and multiplexed assays agree and disagree

Corresponding Author: Professor Joseph Marsh

This file contains all reviewer reports in order by version, followed by all author rebuttals in order by version.

Version 0:

Reviewer comments:

Reviewer #1

(Remarks to the Author)
Why variant effect predictors and multiplexed assays agree and disagree

Livesey and Marsh present a comparative analysis of Computational Variant Effect Predictors (VEPs) and Multiplexed Assays of Variant Effect (MAVEs) in assessing the functional impact of genetic variants, particularly focusing on missense mutations. Authors evaluated 37 MAVE datasets against five state-of-the-art VEPs to quantify and explain areas of agreement and, more significantly, discordance between the two methods. Key findings indicate that VEPs, which heavily rely on sequence conservation, often overcall pathogenicity in buried or hydrophobic regions but undercall impact in disordered or charged surface regions, where MAVEs are often more accurate. Conversely, MAVEs are limited by experimental noise and the assay design, which may fail to capture all disease-relevant mechanisms, such as splicing or gain-of-function mutations, prompting the authors to advocate for integrated, mechanism-aware approaches to improve variant interpretation.

The paper is fairly well-written. Please find my comments below.

1: Throughout the paper, the authors have proposed that VEPs and MAVEs are two different tools for assessing functional consequences of variants. Examples include: line # 11 in Abstract, lines 91-93 (Can we really treat functional measurements from a MAVE and functional predictions from a VEP as independent of each other if they are both attempting to estimate the same value?), etc.

I personally disagree with this notion that VEPs assess function. VEPs predict pathogenicity (as an “effect”), which could be attributed to functon(i.e., activity, binding, stability), constraints, etc. There is another term called “variant function predictors/prediction”, which predicts the function – such as, loss-of-function, gain-of-function, or neutral, and these are not equivalent to “pathogenicity” prediction. MAVEs, depending on the assay, can measure function in a particular dimension, such as VAMP-seq assesses stability, not activity.

Given the importance of the terminologies, I discourage using confusing terms and call VEP to predict pathogenicity and MAVEs to measure function.

2: Line 76 says “true” measurement?? – I am not sure what the authors mean by “true”. I’d replace it with “experimental” measurement.

3: Line 191: “this” figure? Which figure? Authors should refer to the exact figure number. This comment holds for line #400, where the authors say “this” plot. This correction should be applied to the entire manuscript.

4: Line 194-195: The data point for PPARG should be labeled in Figure S2

5: Figure 1: increase the font of panel titles and x and y axes labels

6: Figure 2: all font sizes should increase. Protein feature texts are not legible on (D).

7: Line 911-912 sentence seems to be incorrect. It uses two MAVE in the sentence; the last one should be VEP?

8: Figure 4 Legend (Line 921-922) "The proportion of variants from the VEP-pathogenic, concordant and MAVE-pathogenic classes..." The text defines these sets as VEP-damaging and MAVE-damaging (L260/261), but the Figure 4 legend uses VEP-pathogenic and MAVE-pathogenic. Ensure consistent terminology throughout the text and figures.

9: Authors use five VEPs in the paper, but didn't discuss the variability across them, except in Figure 1 and related texts. Figure 1 shows quite a variability across these VEPs for genes such as GCK, PAX6, and TP53. The paper would be enriched by revising Figure 4 analysis to include data per VEP (VEP-damaging for each of the five VEPs) and MAVE.

10: Lines 573-579 read out of sync with the previous paragraph, which was still on ASPA and PRKN. Then Lines 573-579 talk about VEPs failure due to their assumptions about conservation and go back to SHOC2 and BRCA1! After that, lines 580-593 again talk about experimental noise in MAVEs and take readers back to ASPA and PRKN. The entire text of lines 573-579 reads like a discussion and could be moved there.

Reviewer #2

(Remarks to the Author)
The manuscript, "Why variant effect predictors and multiplexed assays agree and disagree," focuses on where five computational methods and functional assays disagree as a function of protein context across 37 human proteins, while previous works typically assess average VEP to MAVE correlations without further analysis. The identified patterns, e.g. VEPs tending to call variants damaging if in buried cores, at interfaces or in transmembrane helices while MAVEs more often call variants damaging on surface, loops or in IDRs, are informative for method development, protein therapeutics design and clinical interpretation. With tighter analysis and reporting, the study will serve as a practical reference for deciding when to use VEPs, when specific MAVE assays are necessary and when to expect disagreement. Overall, their analsysis supports the principal claims through careful normalization and statistical tests to allow for fair comparison across datasets and reduce circularity and bias arising from clinical label evaluations. The methodology is sound and would be relatively easy to reproduce. In sum, the findings would be useful across multiple fields.
However, some choices may bias the results or hide other useful insights as the study selects a single MAVE per protein based on prior correlation to VEPs and only a handful of VEPs are assessed. The work would be improved by expanding to include more widely employed VEPs, such as REVEL, EVE and PrimateAI-3D, and a larger set of MAVEs. My verdict is publish with revisions: the mapping of where VEPs and MAVEs diverge is valuable to a variety of fields and applications and will be stronger when existing issues are addressed.

Major comments
1. MAVE scope and selection clarity. Please explain why only 37 MAVEs are used given broader availability (for example the 200+ available via Protein Gym). Please either expand the set or provide principled inclusion and exclusion rules that cover duplication, assay class, quality control and clinical relevance.
2. Single assay per gene. Selecting the MAVE that correlates best with VEPs may change the observed discordance, possibly hiding interesting signal from the most discordant MAVEs, especially if distinct functions are assayed for the same protein.
2.1. Please report between assay agreement for proteins with multiple MAVEs, if not in the main text, the supplement.
2.2. Rerun the key analyses using the union of assays and also by stratifying results by assay class, such as VAMP seq vs activity or complementation assays, to see if signal for the specific protein contexts identified survives.
3. VEP selection.
3.1. I would like further clarification on why only 5 VEPs are selected. It is useful to select representative models, but the same analysis would be useful across the top performing VEPs. In particular there are VEPs, such as popEVE, that claim to reduce the pervasive over pathogenicity calling. It would be interesting to see how concordance with MAVEs has changed with the current advances in variant effect predictors.
3.2. In addition, it would be useful to compare to even simpler baseline VEP methods such as PSSMs and to see if they also have the same biases
3.3. Please clarify why SIFT and Polyphen-2 are redundant and therefore not used.
3.4. VARITY fails to have coverage for ~70% of possible substitutions. Are there any other clinical-trained predictors that provide predictions for every possible substitution that could be used instead of VARITY to improve coverage and pooled comparisons?
3.5. An additional analysis that looks for any systematic patterns when there is discordance between the VEPSs would be great.
4. Transmembrane interpretation. From what I understand, mutations in TM helices can interfere with membrane localization into the membrane and thus would have effects on protein function in the cell. However, the authors claim that mutations the TM regions should be far less deleterious than core mutations in globular proteins as it should not interfere with the usual protein folding process.
4.1. Can you please expand on the comments made about the expected fitness effects of mutations in transmembrane helices? The conclusion that MAVEs provide a more "balanced" view of the effects of TM mutations does not seem to be fully supported.
4.2. How different are the distributions of VEP scores for internal residues versus transmembrane residues? Are they actually predicted to be as deleterious? Do the MAVEs also predict both internal and transmembrane residues to be similarly benign or non-pathogenic?
5. Why MAVEs and VEPs disagree.

5.1. Please clarify statements about MAVEs providing a more balanced overview of variant fitness. Is this a quantitative thing where VEPs may have more bimodal distributions while MAVE outputs are smoother or is this qualitative?
5.2. Is it undercalling if the mutations are phenotypically neutral? “In other words, VEPs are likely overcalling substitutions that are typically considered the most damaging and undercalling those that are most often phenotypically neutral while MAVEs are producing a more balanced overview.”
5.3. Do these assumptions hold true for VEPs that have models that are built for each individual protein sequence and not directly applied to other proteins?
5.3.1. “VEPs have to make assumptions (during their training process) about the types of variants that would typically be damaging in order to be generalisable across many different proteins.”
5.3.2. “MAVEs are unique for each protein, leading to a larger range of predictions at each position that is unconstrained by prior assumptions of residue-level mutational tolerance.”
5.4. Are any of the identified residue classes enriched for ClinVar pathogenic variants? For example, do ClinVar path variants tend to be in the protein interior or interface regions or do they tend to be at the surface? Do these pathogenic variants tend to be bulky/hydrophobic or more charged/polar?
5.5. Do MAVEs that assess protein stability and folding agree more often with VEPs than those that assess a specific function?

Minor comments
6. Circularity and population bias. The manuscript states that selected population-tuned VEPs are immune or highly resistant to circularity.
6.1. Please add citations for this sentence in particular: “We previously found each of these methods to be either immune or highly resistant to the phenomenon of data circularity, so making comparisons using annotated disease variants should not materially affect the results”.
6.2. I would be interested to see if MAVEs fare better than population-tuned VEPs if the gnomad benign set were derived solely from individuals of non-European ancestry.
6.3. Please clarify whether gnomAD neuro and cancer cohorts are excluded when constructing the putatively benign set representing the generally “healthy” population.
7. Disorder definition and alternatives.
7.1. Please, either justify the use of pLDDT with citations or complement it with an orthogonal IDR caller such as IUPred or Metapredict.
7.2. I would also like to see the counts and effect sizes within predicted IDRs compared to ordered regions. Are MAVEs identifying the handful of variants in IDRs that are truly deleterious or do their scores on average skew deleterious in IDRs?
7.3. Additionally, it would be useful to individually assess-based and alignment-free VEPs as their performance in IDRs may differ significantly.
8. Case examples.
8.1. For SNCA, I would argue that rather than the length of the human lifespan, these mutations would not be seen as pathogenic as their effects would only be seen post-reproductive age, which may be true across other species as well. Further, its to be expected that VEPs trained on conservation across the evolutionary tree would not be able to detect signal from late-onset diseases as well as MAVEs.
8.2. For BRCA1, do all state-of-the-art VEPS beyond the five assessed call the three example mutations as benign? Does concordance for BRCA1 and across all other MAVEs improve if predicted splice variants are filtered out?
8.3. For SHOC2, where are the pathogenic mutations, other than S2G, in the protein sequence? Arguably, alignment-based methods will perform poorly at assessing mutations at the beginning or end of a protein sequence as alignment coverage is poor in these regions, rather than being a consequence of the VEPs failing to identify a rare gain-of-function mutation.
9. Minor figure issues and requests.
9.1. Add uncertainty and formal tests wherever differences are reported. Bar charts should include error bars or confidence intervals. Show the distribution of Spearman correlations per VEP and per MAVE rather than only means. Include VEP versus VEP and MAVE versus MAVE disagreement maps to show internal dispersion.
9.2. Please add residue classification (i.e. hydrophobicity) to the marginals of Figure 3.
9.3. For RSA and pLDDT differences in Figure 4, please add error bars or CIs to the bar charts if applicable and statistics to see if differences between the concordance classes are significant.
9.4. For Figure 6, please add the distributions to the marginals for each clinical label class and gene.
9.5. Figure S1, please use a perceptually uniform colorway instead of Jet (https://gorelik.net/2020/08/17/what-is-the-biggest-problem-of-the-jet-and-rainbow-color-maps-and-why-is-it-not-as-evil-as-i-thought).
9.6. Expansion on Figure S1 – what are the distributions of Spearman’s across for each VEP across all MAVEs?

Reviewer #3

(Remarks to the Author)
Summary:

Livesey and Marsh ask why variant effect predictors (VEPs) and multiplexed assays of variant effect (MAVEs) differ and offer insights through analysis across 37 MAVEs and 5 state-of-the-art VEPs. This question is extremely important and timely as new recommendations for the classification of genetic variants are being drafted by ClinGen, and as the authors note in the introduction, the clinical community is struggling with the question: “Can we really treat functional measurements from a MAVE and functional predictions from a VEP as independent of each other if they are both attempting to estimate the same value?” To help answer this question the authors detect where MAVEs and VEPs disagree and offer explanations for the cause of this discrepancy. For example, MAVEs are enriched for deleterious measurements at disordered regions compared

to VEPs and VEPs tend to overcall deleteriousness at buried regions compared to MAVE measurements. These patterns are consistent with individual MAVE analyses and offer a broader look across many assays. The authors point to specific variants with explainable discordance across 9 proteins and conclude that the combination of MAVE and VEP are ultimately poised to better reflect true fitness effects of variants than either method individually.

Major comments:

1. My biggest critique of the manuscript is the use of normalized rank scores for the MAVE datasets. While I agree with the authors that MAVE data presents a challenge for direct comparison with most VEPs due to different scoring methods and scales, the normalized rank scores compress assay dynamic range and cause wild type-like/functionally normal variants to score like deleterious variants. While this normalization should not impact AUROC values in Figure 1, it can influence each of the subsequent analyses. The problem with ranked scores is that most MAVE scores are wild type-like/functionally normal and assigning ranks to functionally normal variants might artificially cause signal from assay noise. For example, in Figure 2C the MAVE more pathogenic plot might be reflecting wild type-like scores as more pathogenic. We can see from the positional ranked plot in Figure 2A that the region of amino acids 200-250 does not have any variants that rank as high as the positions from amino acids 50-150 and these likely represent functionally normal variants, yet they are called MAVE more pathogenic. A min-max 0-1 normalization should avoid such effects and move many of these variants to the middle of the score distribution while more accurately identifying the rare positions that are MAVE more pathogenic. We can also visualize this effect in the analysis of the MSH2 MAVE from Jia et al. where ~90% of the variants have functionally normal scores and are distributed like synonymous variants. However, ranking variant scores gives many of these wild type-like variants high scores and can have consequences when comparing to VEP outputs, making the MAVE appear to call more variants deleterious and fewer variants wild type-like than is actually reflected in the data. This effect can be visualized in Figure 6 where many of the ClinVar/gnomAD benign MSH2 scores are to the right side of the plot and relatively few are at the top left. Finally, this score normalization also impacts the wide positional score range the authors report for MAVEs. Although this pattern is not unexpected, it can be exaggerated with rank normalization. While the overall patterns that the authors detect are consistent with reported MAVE and VEP concordance/discordance from other studies, the magnitudes of discordance will likely be shifted across many of the genes. This effect might not be as dramatic for VEP score normalization, but the loss of distribution shapes will have an impact on the analysis and I recommend considering a different normalization procedure that maintains distribution shape.

2. Additionally, the authors could provide more clarity around assay selection. I thought the introduction to the VEPs and rationale for choosing the five VEPs for comparison was very clear and helpful for setting up and understanding the analysis. However, the MAVE selection was not as clear and as a result, the interpretability of the findings are less clear. For example, the authors could consider breaking down MAVE selection in a similar way to VEPs, but by assay type. MAVEs can be grouped by model system (yeast vs. human cells), by variant integration method (SGE vs. viral integration vs. plasmid), and by functional readout (cell survival vs. protein abundance vs. reporter). Each of these classes has strengths and weaknesses and grouping by class may reveal important distinctions. For example, the authors point out that SGE is typically more accurate than transgene approaches since the SGE cell viability phenotype can be agnostic to variant pathomechanism. However, only 2 SGE datasets are included in the analysis while many more quality SGE datasets have been published in the last few years. For example VHL (PMID: 38969834), TP53 (PMID: 39774325), BRCA2 (PMID: 39779848), DDX3X (PMID: 38057330) and BAP1 (PMID: 38969833) all have accurate SGE datasets which may be important to consider for this analysis. These assays are especially important to consider for the clinical evidence weight problem as SGE assays typically achieve the strongest evidence strength so at minimum, their inclusion in this analysis would be helpful. Ideally, breaking up the analysis by MAVE method would yield the most impactful insights for the clinical community.

3. Additionally, while the authors provide sound justification for the predictors they choose for the analysis, the two most commonly used predictors in variant classification (REVEL and BayeDel) are left out. Since the rationale for the analysis is built around the use of MAVE and VEP for variant classification, these two should at least be discussed in the main text/discussion. I agree with the authors that these methods are vulnerable to circularity, but some discussion on how this might impact the analysis would also be helpful.

Minor comments:

1.Readers likely know this but I suggest adding the word “pooled” in the description of MAVEs on page 2. High throughput could also refer to arrayed experiments that are not technically MAVEs.

2. Typical assay description should add “transfect” after transform to indicate assays in both yeast and human cells.

3. This sentence is not technically true since the Pejaver et al paper referenced in the next sentence represents the updated ClinGen recommendation: “Currently VEPs are only capable of providing supporting evidence for pathogenicity while functional assays such as MAVEs can provide up to strong evidence.” I think it is fine to change this to “Historically, VEPs were…”

4. Figure 3 legend typo: “MAVEs fund” to “MAVEs find”.

5. Use of "fitness assay” to describe VAMPseq on page 9 and Table 1 is confusing since VAMPseq is not technically a cellular fitness assay. I suggest just using protein abundance/stability instead.

Version 1:

Reviewer comments:

Reviewer #1

(Remarks to the Author)
I approve of the revised manuscript. I appreciated the clarification of terms - what VEPs and MAVE measure, the inclusion of more MAVE sets, and more VEPs.

Reviewer #2

(Remarks to the Author)
The authors have sufficiently addressed all of my concerns. I consider this paper ready for publication.

Reviewer #3

(Remarks to the Author)
In their revised submission, Livesey and Marsh made significant updates to the analysis and description of results in their manuscript. Updates include broadening both the VEPs (to the top 5 performing population aware and top 5 population free models) and the MAVEs (99 datasets across 47 MAVEs with principled inclusion criteria) offering a more robust view of the agreement and disagreement between the two sources of data. The authors also group MAVEs by type of selection which offers more insight into which methods may agree more than others. In addition, the normalization of VEP and MAVE scores were adjusted to z-score normalization which maintains distribution shape better than rank normalization. Some new compelling results include: patterns of agreement and disagreement are broadly consistent across assay types, suggesting significant overlap in the pathomechanisms detectable by MAVEs. Additionally, variants in DNA-binding domains appear to be consistently over-predicted as deleterious across VEPs highlighting a potential area of consistent bias across model training. These changes satisfy my original concerns about the analysis and add greater nuance to the results and discussion that will be of value to the field and should be carefully considered by groups developing frameworks for the combination of these data sources for clinical variant classification. I have several minor comments to be considered before publication:

Minor comments:

1) Intro line 94: “40 MAVE datasets” should be 99 scoreset from 47 MAVE studies, or simply MAVE data from 40 human proteins.

2) Line 141: “Although some widely used clinical meta-predictors such as REVEL and BayesDel were not included, the expanded clinical-trained set is intended to provide representative coverage of this predictor class.” After reading the author’s response to reviews and the additional analysis of within class VEP differences, I agree with the rationale of excluding these predictors. However, I do think this sentence is out of place and does not offer readers the same context that we have as reviewers. As the authors mention in their response: “Our focus is on the broad differences between VEPs and MAVEs, rather than on fine-grained distinctions among individual clinical-trained predictors. Given the substantial agreement observed among VEPs within this class, we do not expect inclusion of REVEL or BayesDel to materially alter the main conclusions.” I think it would be better to move the description of the exclusion of REVEL and BayesDel to the paragraph starting at line 159 to give the readers the full context of their exclusion.

3) Line 173: The separation of MAVEs by type of selection is a great value add to the manuscript. However, the result for performance of fitness based assays is unexpected as SGE in HAP1 cells has been the gold standard for clinical performance amongst MAVEs. Only after looking into the supplement is it clear that most fitness assays are non-endogenous editing and non-human cell line assays. While I do not have a problem with grouping assays in this way, it would be helpful to the audience to make this distinction within the group since this will be the overwhelming expectation from MAVE and clinical classification scientists. I think this is important to state because it may distract readers from the extremely important conclusion: “This implies a degree of consistency across MAVE datasets despite differences in assay design, with broad patterns of agreement and disagreement with VEPs largely preserved”.

4) The SNCA A53T example is confusing. I understand that the low z-score for the population-free VEPs suggests that those predictors consistently call this variant tolerated, but the z-score around zero for the other VEPs and MAVE suggest that they also fail to detect the true pathogenicity of this variant. The interpretation of the z-scores across each dataset in the context of SNCA variants needs to be clarified in this section.

5) MSH2 S612P is a very interesting example where VEPs may outperform the MAVE, but stating that the ClinVar assessment is likely wrong is a bit of a stretch. This claim should either be backed up by additional evidence (beyond population frequency) or the wording should be hedged significantly. Just as we wouldn’t call a variant pathogenic based on a single piece of evidence (in most cases), we should not assume the interpretation is wrong based on the VEP evidence. The VEP evidence is compelling in this case for sure, but since we do not know all of the evidence that Ambry used in this classification, I would stop short of suggesting their interpretation is incorrect.

6) I disagree with the assessment of the CARD11 C49Y variant. While the z-score normalization is a reasonable approach for comparing scores, the interpretation of assay scores would function differently in practice. The MAVE community has adopted language of functionally normal vs functionally abnormal and these distinctions are usually made by the distribution of synonymous variants in the assay. Since the C49Y variant is significantly enriched in the CARD11 fitness assay it would both have a high z-score relative to the synonymous distribution and would be called functionally abnormal. In this sense the assay is actually concordant with the predictors which yield consistent deleterious predictions. This is still a useful example of a caveat to the analysis used in this manuscript, but this would not be considered discordant in variant classification practice. The authors should amend the text to reflect this nuance.

Open Access This Peer Review File is licensed under a Creative Commons Attribution 4.0 International License, which permits use, sharing, adaptation, distribution and reproduction in any medium or format, as long as you give appropriate credit to the original author(s) and the source, provide a link to the Creative Commons license, and indicate if changes were made.
In cases where reviewers are anonymous, credit should be given to 'Anonymous Referee' and the source.
The images or other third party material in this Peer Review File are included in the article's Creative Commons license, unless indicated otherwise in a credit line to the material. If material is not included in the article's Creative Commons license and your intended use is not permitted by statutory regulation or exceeds the permitted use, you will need to obtain permission directly from the copyright holder.
To view a copy of this license, visit https://creativecommons.org/licenses/by/4.0/

Response to reviewers

Reviewer comments are reproduced verbatim in shaded italic text, with our responses directly following each relevant point. Text added to the revised manuscript is shown in quotation marks and italics.

Reviewer #1 (Remarks to the Author):

Why variant effect predictors and multiplexed assays agree and disagree

Livesey and Marsh present a comparative analysis of Computational Variant Effect Predictors (VEPs) and Multiplexed Assays of Variant Effect (MAVEs) in assessing the functional impact of genetic variants, particularly focusing on missense mutations. Authors evaluated 37 MAVE datasets against five state-of-the-art VEPs to quantify and explain areas of agreement and, more significantly, discordance between the two methods. Key findings indicate that VEPs, which heavily rely on sequence conservation, often overcall pathogenicity in buried or hydrophobic regions but undercall impact in disordered or charged surface regions, where MAVEs are often more accurate. Conversely, MAVEs are limited by experimental noise and the assay design, which may fail to capture all disease-relevant mechanisms, such as splicing or gain-of-function mutations, prompting the authors to advocate for integrated, mechanism-aware approaches to improve variant interpretation.

The paper is fairly well-written. Please find my comments below.

1: Throughout the paper, the authors have proposed that VEPs and MAVEs are two different tools for assessing functional consequences of variants. Examples include: line # 11 in Abstract, lines 91-93 (Can we really treat functional measurements from a MAVE and functional predictions from a VEP as independent of each other if they are both attempting to estimate the same value?), etc.

I personally disagree with this notion that VEPs assess function. VEPs predict pathogenicity (as an "effect"), which could be attributed to functon(i.e., activity, binding, stability), constraints, etc. There is another term called "variant function predictors/prediction", which predicts the function – such as, loss-of-function, gain-of-function, or neutral, and these are not equivalent to "pathogenicity" prediction. MAVEs, depending on the assay, can measure function in a particular dimension, such as VAMP-seq assesses stability, not activity.

Given the importance of the terminologies, I discourage using confusing terms and call VEP to predict pathogenicity and MAVEs to measure function.

Response: We have substantially revised the manuscript to clarify the distinction between what VEPs and MAVEs quantify. Rather than describing both as measuring "function", we now refer more generally to their assessment of variant impact. MAVEs typically measure the effect of variants on a specific experimental readout (e.g. cellular fitness, protein activity, or abundance), whereas VEPs estimate a broader notion of variant impact, often derived from evolutionary constraint and structural context. Where appropriate, we use the term "damaging" as a general shorthand for variant effects that adversely affect molecular function or fitness, or increase the likelihood of pathogenicity, while recognising that these are related but not equivalent concepts. We have added a paragraph in the Introduction to make this distinction clearer.

> *"There is an important distinction in what MAVEs and VEPs quantify, which raises questions about how directly their scores can be compared. MAVEs typically measure the effect of variants on a specific experimental readout, such as protein stability, activity, or abundance, depending on the assay design. By contrast, VEPs generally estimate a broader notion of variant impact, often derived from evolutionary constraint, sequence context, and structural features. While these scores are frequently used as proxies for pathogenicity, many modern VEPs are not explicitly trained to predict disease, but instead capture more general properties related to fitness or constraint. As a result, similar scores from a MAVE and a VEP do not necessarily reflect the same underlying quantity. For example, a variant may strongly disrupt a particular molecular function without causing disease, or conversely may have near wild-type activity in a given assay but still be pathogenic through mechanisms not captured by that assay. Some MAVEs measure cellular fitness rather than a specific biochemical function, which may align more closely with the signals captured by VEPs, and conversely some predictors aim to estimate more specific properties such as protein stability. Despite these differences, both approaches ultimately attempt to capture the consequences of genetic variation, albeit at different levels and with different assumptions."*

2: Line 76 says "true" measurement?? – I am not sure what the authors mean by "true". I'd replace it with "experimental" measurement.

Response: We no longer refer to MAVEs as representing "true" measurement.

3: Line 191: "this" figure? Which figure? Authors should refer to the exact figure number. This comment holds for line #400, where the authors say "this" plot. This correction should be applied to the entire manuscript.

Response: All references to figures and tables now explicitly state which figure (and sub-panel) they are referring to.

4: Line 194-195: The data point for PPARG should be labeled in Figure S2

Response: Due to a substantial re-work (in response to comments from reviewers 2 and 3) this figure has been changed to a boxplot (still as Figure S2) due to the increase in dataset size making a scatter plot more cluttered. The choice was also made since there was little value gained by assessing standard deviation in a small window and per-position simultaneously. PPARG is no longer remarked on in the text at this point.

5: Figure 1: increase the font of panel titles and x and y axes labels

Response: Figure 1 has been re-worked substantially and now has larger text sizes relative to the figure.

6: Figure 2: all font sizes should increase. Protein feature texts are not legible on (D).

Response: All axes ticks, axes labels and legend text sizes have been increased substantially in Figure 2. Please note that the protein assessed in Figure 2 has changed from LDLRAP1 to PAX6 due to LDLRAP1 now being excluded (see response to reviewer 2). The figure has also been streamlined. Panel D now shows MAVE and VEP trends across the protein individually rather than a single difference score. The former panel C has been removed and panel E now shows a mapping of the difference score on to the 3D protein structure.

The changes in figure text size have also been applied to the other protein feature plots in the paper (Figures 5 and S6).

7: Line 911-912 sentence seems to be incorrect. It uses two MAVE in the sentence; the last one should be VEP?

Response: Figure 2 has been substantially changed and no longer shows the difference score; this wording is no longer present in the legend.

8: Figure 4 Legend (Line 921-922) "The proportion of variants from the VEP-pathogenic, concordant and MAVE-pathogenic classes..." The text defines these sets as VEP-damaging and MAVE-damaging (L260/261), but the Figure 4 legend uses VEP-pathogenic and MAVE-pathogenic. Ensure consistent terminology throughout the text and figures.

Response: The concept of using VEP-damaging and MAVE-damaging variants has been fully removed from the manuscript. This has been replaced with a correlation-based analysis on a per-dataset basis to various protein attributes (Figure 4). The wording in the figure legends has been checked for consistency with the main text.

9: Authors use five VEPs in the paper, but didn't discuss the variability across them, except in Figure 1 and related texts. Figure 1 shows quite a variability across these VEPs for genes such as GCK, PAX6, and TP53. The paper would be enriched by revising Figure 4 analysis to include data per VEP (VEP-damaging for each of the five VEPs) and MAVE.

Response: The analyses are now split between two groups of representative VEPs to help show the variance in VEP performance slightly more.

All VEPs are now compared in Figure S1, and their correlations across all MAVE sets is available in Supplementary Data 2. Additionally, a comparison between the two groups of VEPs is performed in Supplementary Data 6 for annotated protein features. All VEP scores are available for ClinVar variants in Supplementary Data 7, and some differences are remarked on in the text:

> *"A study also found that D1818G impacts splicing and results in skipping of exon 22. Nine of the ten VEPs in the analysis generate negative z-scores for these variants, the exception is SNPred, which produces positive z-scores for the two variants at A1823."*

We also note that we have now released a preprint further investigating the agreement and disagreement across VEPs (https://www.biorxiv.org/content/10.64898/2026.03.18.712765v1), which we now mention in the text:

“A related question, which we have not explored in detail here, is the extent to which different VEPs agree with one another. While our analysis focuses on the relationship between VEPs and MAVEs, inter-predictor variability is substantial and may itself be informative. Recently, we systematically examined concordance across multiple VEPs at proteome scale and found that agreement varies widely across proteins, reflecting differences in model features and training paradigms rather than intrinsic predictability. Notably, inter-VEP agreement showed little relationship with agreement to experimental data, suggesting that predictor consensus should not be interpreted as a proxy for accuracy. Instead, regions of disagreement may highlight mechanistic blind spots and provide a rationale for prioritising experimental follow-up.”

> *10: Lines 573-579 read out of sync with the previous paragraph, which was still on ASPA and PRKN. Then Lines 573-579 talk about VEPs failure due to their assumptions about conservation and go back to SHOC2 and BRCA1! After that, lines 580-593 again talk about experimental noise in MAVEs and take readers back to ASPA and PRKN. The entire text of lines 573-579 reads like a discussion and could be moved there.*

Response: We have revised the “Analysis of discordantly predicted variants” section so that each protein is discussed only within its own section. The points about VEP and MAVE failures was mostly redundant with the material in the discussion section, and so has been merged with that or removed.

Reviewer #2 (Remarks to the Author):

> *The manuscript, “Why variant effect predictors and multiplexed assays agree and disagree,” focuses on where five computational methods and functional assays disagree as a function of protein context across 37 human proteins, while previous works typically assess average VEP to MAVE correlations without further analysis. The identified patterns, e.g. VEPs tending to call variants damaging if in buried cores, at interfaces or in transmembrane helices while MAVEs more often call variants damaging on surface, loops or in IDRs, are informative for method development, protein therapeutics design and clinical interpretation. With tighter analysis and reporting, the study will serve as a practical reference for deciding when to use VEPs, when specific MAVE assays are necessary and when to expect disagreement. Overall, their analsysis supports the principal claims through careful normalization and statistical tests to allow for fair comparison across datasets and reduce circularity and bias arising from clinical label evaluations. The methodology is sound and would be relatively easy to reproduce. In sum, the findings would be useful across multiple fields.*
> *However, some choices may bias the results or hide other useful insights as the study selects a single MAVE per protein based on prior correlation to VEPs and only a handful of VEPs are assessed. The work would be improved by expanding to include more widely employed VEPs, such as REVEL, EVE and PrimateAI-3D, and a larger set of MAVEs. My verdict is publish with revisions: the mapping of where VEPs and MAVEs diverge is valuable to a variety of fields and applications and will be stronger when existing issues are addressed.*

Major comments

> *1. MAVE scope and selection clarity. Please explain why only 37 MAVEs are used given broader availability (for example the 200+ available via Protein Gym). Please either expand the set or provide principled inclusion and exclusion rules that cover duplication, assay class, quality control and clinical relevance.*

Response: We thank the reviewer for this suggestion. In response, we have systematically expanded and re-curated the MAVE dataset by surveying all available studies in MaveDB, ProteinGym, and additional datasets suggested by Reviewer 3. Rather than selecting a subset, we applied a consistent set of inclusion and exclusion criteria across all available datasets to define a high-quality and comparable set for analysis.

Specifically, we retained only MAVEs that: (i) report scores for at least 1,000 single amino acid substitutions, (ii) are accompanied by a publication or documentation clearly describing the assay methodology, and (iii) have sufficient overlap with clinical data, defined as at least 5 pathogenic or likely pathogenic variants in ClinVar. In addition, we excluded datasets from extremely large-scale studies (e.g. Beltran et al., 2025; Tsuboyama et al., 2023) to avoid over-representation of results generated under highly similar experimental conditions, particularly protein stability assays.

Applying these criteria across all available datasets resulted in a final set of 99 score sets from 47 studies covering 40 unique human proteins. While this number is smaller than the total number of MAVEs available, many datasets do not meet these criteria, most commonly due to limited variant coverage, lack of sufficient clinical overlap, or redundancy in assay type. The revised dataset, along with the full list of included studies (Supplementary Data 1), is now described in the Results section, and all analyses have been updated accordingly.

2. Single assay per gene. Selecting the MAVE that correlates best with VEPs may change the observed discordance, possibly hiding interesting signal from the most discordant MAVEs, especially if distinct functions are assayed for the same protein.

Response: To address this concern, we have revised the analyses of protein-level properties, domain annotations, and specific variants to include all 99 MAVE assays independently, rather than selecting a single assay per protein. This allows the full range of assay-specific behaviour, including potentially discordant signals, to be retained and visualised.

For the amino acid-level analysis, applying this approach was not feasible due to the increased sparsity and complexity of the data when aggregating across all assays. For this analysis, we therefore retained a single representative assay per protein, but revised the selection criterion to use the highest ROC AUC rather than correlation with VEPs, avoiding potential bias toward concordance.

In addition, to assess the robustness of the results to assay type, we stratified MAVEs by assay class and repeated the amino acid-level analysis within these subsets (Figure S3).

2.1. Please report between assay agreement for proteins with multiple MAVEs, if not in the main text, the supplement.

Response: The correlation between MAVE assays is now discussed following Figure S1 and compared to the MAVE/VEP correlations. The comparison is available in Supplementary Data 3.

> *"For the 18 proteins where multiple MAVEs were available, the mean correlation between assays was 0.61 (Supplementary Data 3)."*

2.2. Rerun the key analyses using the union of assays and also by stratifying results by assay class, such as VAMP seq vs activity or complementation assays, to see if signal for the specific protein contexts identified survives.

Response: We have re-run the amino acid substitution analysis using MAVEs with different assay classes (Figure S3).

> *"To investigate whether the relationship between MAVEs and VEPs at specific amino acid substitutions is influenced by the MAVE methodology, we repeated the analysis by sub-setting the MAVE dataset by assay type. Our findings (Figure S3) show a generally similar pattern of MAVE/VEP disagreement for each of the assay types, although the pattern becomes less clear for stability and trafficking assays, possibly due to the lower number of MAVE assays in these groups. This implies a degree of consistency across MAVE datasets despite differences in assay design, with broad patterns of agreement and disagreement with VEPs largely preserved. Differences between assays are therefore more likely to be evident at the level of specific protein features or individual variants."*

As mentioned in response to a previous point, other key analyses are now run using all MAVE datasets rather than a subset.

3. VEP selection.

3.1. I would like further clarification on why only 5 VEPs are selected. It is useful to select representative models, but the same analysis would be useful across the top performing VEPs. In particular there are VEPs, such as popEVE, that claim to reduce the pervasive over pathogenicity calling. It would be interesting to see how concordance with MAVEs has changed with the current advances in variant effect predictors.

3.2. In addition, it would be useful to compare to even simpler baseline VEP methods such as PSSMs and to see if they also have the same biases

Response: We have now expanded our VEP set to include 10 representative predictors, comprising 5 population-free and 5 clinical-trained methods, selected based on performance in recent benchmarks (Livesey & Marsh, 2025). This provides broader coverage of current state-of-the-art approaches while maintaining a balance of methodological diversity.

Importantly, we find that VEPs show substantially higher agreement with one another than with MAVE data (Figure S1). This high level of inter-VEP concordance suggests that adding additional predictors does not materially change the observed patterns of discordance with MAVEs, but instead reinforces the shared behaviour across VEPs. As a result, our focus is on the systematic differences between computational predictions and experimental measurements, rather than on fine-grained differences between individual predictors.

To address the reviewer's suggestion regarding simpler baseline methods, we also assessed a substitution-based baseline (BLOSUM62). This shows a broadly similar pattern of discordance with MAVE data, supporting the conclusion that evolutionary conservation is a major contributor to the observed differences. This analysis is now included in Figure S5a.

3.3. Please clarify why SIFT and Polyphen-2 are redundant and therefore not used.

Response: We have removed the statement suggesting that methods such as SIFT and PolyPhen-2 are "redundant", as we agree that this was not an appropriate characterisation.

In the revised manuscript, we instead define a clear and principled selection strategy, choosing 10 representative VEPs (5 population-free and 5 clinical-trained) based on performance in recent benchmarks (Livesey & Marsh, 2025). Our aim is to capture the behaviour of current state-of-the-art predictors rather than to exhaustively compare all available methods.

Methods such as SIFT and PolyPhen-2 were not included because they do not meet these selection criteria and have been consistently outperformed by more recent approaches in independent evaluations. However, as noted in the manuscript, the strong agreement observed across diverse modern VEPs suggests that our conclusions are not sensitive to the inclusion or exclusion of specific individual predictors.

3.4. VARITY fails to have coverage for ~70% of possible substitutions. Are there any other clinical-trained predictors that provide predictions for every possible substitution that could be used instead of VARITY to improve coverage and pooled comparisons?

Response: By their nature, clinical-trained methods are almost always limited to single nucleotide variants because these variants solely comprise their training data. We do, however agree that having a single predictor with lower coverage has the potential to skew aspects of the analysis. This was a major factor in deciding to include clinical-trained predictors as a separate predictor category, which allows VARITY to be grouped with other VEPs that have the same pattern of predictions. This decision also allows comparison to be made between the two predictor groups.

3.5. An additional analysis that looks for any systematic patterns when there is discordance between the VEPSs would be great.

Response: A full all vs all analysis across all of the VEP is unrealistic due to the high number of combinations. To simplify this, we elected to perform the comparison between the population-free and clinical trained VEP sets, similarly to how MAVE and VEP data were compared. We repeated the analysis across protein-level annotations to identify where the two groups of VEPs demonstrated the greatest discordance (Supplementary Data 6).

> *"We also investigated discordance between population-free and clinical-trained VEPs. For each annotated region, we calculated the difference between the median z-scores of the two groups (Supplementary Data 6), where positive values indicate higher impact scores from population-free VEPs. As with the MAVE comparison, the largest differences occur in disordered regions. However, unlike the MAVE-VEP comparison, the direction of these differences is not consistent, with both positive and negative values observed. The magnitude of the differences is generally smaller than in the MAVE comparison, with notable exceptions including extended disordered regions in MLH1 and VHL. The largest positive differences are primarily observed at binding sites, including zinc-binding residues in TP53, ATP-binding residues in MET, and DNA-contact residues in CRX. Overall, differences between the two VEP groups are substantially smaller than those observed between MAVEs and VEPs, consistent with the generally higher concordance among VEP predictions."*

As mentioned earlier, we also briefly discuss in the text a new preprint further investigating the agreement and disagreement across VEPs (https://www.biorxiv.org/content/10.64898/2026.03.18.712765v1).

4. Transmembrane interpretation. From what I understand, mutations in TM helices can interfere with membrane localization into the membrane and thus would have effects on protein function in the cell. However, the authors claim that mutations the TM regions should be far less deleterious than core mutations in globular proteins as it should not interfere with the usual protein folding process.

4.1. Can you please expand on the comments made about the expected fitness effects of mutations in transmembrane helices? The conclusion that MAVEs provide a more "balanced" view of the effects of TM mutations does not seem to be fully supported.

4.2. How different are the distributions of VEP scores for internal residues versus transmembrane residues? Are they actually predicted to be as deleterious? Do the MAVEs also predict both internal and transmembrane residues to be similarly benign or non-pathogenic?

Response: In revising the dataset using more stringent and clearly defined inclusion criteria, the number of proteins with annotated transmembrane regions was substantially reduced. In the final dataset, only two proteins contain annotated transmembrane regions, and only one (KCNH2) includes a substantial transmembrane domain.

Given this limited representation, we concluded that the dataset is no longer sufficient to support a general analysis or detailed interpretation of variant effects in transmembrane regions. We have therefore removed the extended discussion and associated speculation from the manuscript, and now restrict our comments to brief observations of the discordance present in the remaining data.

5. Why MAVEs and VEPs disagree.

5.1. Please clarify statements about MAVEs providing a more balanced overview of variant fitness. Is this a quantitative thing where VEPs may have more bimodal distributions while MAVE outputs are smoother or is this qualitative?

5.2. Is it undercalling if the mutations are phenotypically neutral? "In other words, VEPs are likely overcalling substitutions that are typically considered the most damaging and undercalling those that are most often phenotypically neutral while MAVEs are producing a more balanced overview."

Response: We agree that the term "balanced" was not clearly defined and could be interpreted as referring to the distribution of scores. Our intention was not to describe differences in score distributions (e.g. bimodality versus smoothness), but rather to highlight systematic differences in how MAVEs and VEPs assign impact to different classes of amino acid substitutions. In particular, VEPs tend to assign higher impact to substitutions that are generally considered deleterious based on evolutionary and physicochemical constraints, whereas MAVEs, which measure variant effects in a specific experimental context, can capture cases where these general expectations do not hold. Conversely, substitutions between residues with similar properties, which are often assumed to be benign, are more frequently assigned higher impact by MAVEs. To clarify this point and avoid misinterpretation, we have revised the relevant text to remove the term "balanced" and instead describe these patterns more explicitly, as shown below.

> *"Mutations involving large physicochemical changes or residues with unique properties are generally considered more likely to be damaging, and these are consistently assigned higher impact by VEPs than by MAVEs. This pattern is consistent with VEPs relying on generalised evolutionary and structural constraints, in which similar substitutions are typically deleterious. These observations are also in line with previous studies showing that pathogenic variants are enriched in conserved, buried, and functionally important residues46–49. By contrast, MAVEs measure variant effects in a specific experimental context and therefore capture cases where these general trends do not hold. Conversely, substitutions between residues with similar properties, which are often assumed to be benign, are more frequently assigned higher impact by MAVEs, suggesting that the impact of mutations at these residues may be underestimated by VEPs."*

5.3. Do these assumptions hold true for VEPs that have models that are built for each individual protein sequence and not directly applied to other proteins?

5.3.1. "VEPs have to make assumptions (during their training process) about the types of variants that would typically be damaging in order to be generalisable across many different proteins."

5.3.2. "MAVEs are unique for each protein, leading to a larger range of predictions at each position that is unconstrained by prior assumptions of residue-level mutational tolerance."

Response: We agree that predictors such as EVE differ from global models in that they are trained separately for each protein and therefore do not need to generalise across proteins. However, these methods still rely heavily on evolutionary information derived from multiple sequence alignments, and so can inherit similar residue-level constraints on substitution preferences.

To address this directly, we included EVE in an additional analysis (Figure S5b). Despite its per-protein modelling approach, EVE shows a broadly similar pattern of discordance with MAVE data to the other VEPs. In the revised manuscript, we now note that this indicates the observed trends are not primarily dependent on cross-protein generalisation, but instead reflect the shared reliance of many VEPs on evolutionary information. We also included BLOSUM62 as a simple substitution-based baseline (Figure S5a), which likewise shows a similar pattern, further supporting this interpretation.

> *"Consistent with this, a simple substitution-based baseline using BLOSUM62 showed a broadly similar pattern of discordance with MAVE data (Figure S5a), supporting the conclusion that evolutionary conservation is a major contributor to these differences. Likewise, EVE55, a per-protein model that learns constraints solely from sequence alignments, shows a similar pattern of discordance with MAVE data (Figure S5b), indicating that these trends are not dependent on cross-protein generalisation but instead reflect shared reliance on evolutionary information."*

5.4. Are any of the identified residue classes enriched for ClinVar pathogenic variants? For example, do ClinVar path variants tend to be in the protein interior or interface regions or do they tend to be at the surface? Do these pathogenic variants tend to be bulky/hydrophobic or more charged/polar?

Response: The distribution of pathogenic variants across structural regions and amino acid classes has been extensively characterised in previous studies, which have consistently shown enrichment in conserved, buried, and functionally important residues. As our focus here is on differences between MAVEs and VEPs rather than re-characterising the general properties of pathogenic variants, we have not performed additional enrichment analyses. However, to acknowledge this prior work, we now explicitly note in the manuscript that our observations are consistent with these established patterns.

> *"These observations are also in line with previous studies showing that pathogenic variants are enriched in conserved, buried, and functionally important residues46–49"*

> *5.5. Do MAVEs that assess protein stability and folding agree more often with VEPs than those that assess a specific function?*

Response: We have added a section to the discussion looking at not only folding/stability MAVEs, but all categories. We do not see any particular increase in agreement at the scale of the full protein, but some differences are observed at the level of protein features and variants. These don't necessarily favour any particular MAVE category, but rather tend to reflect limitations of the specific MAVE.

> *"There was no substantial link between the methodology underlying a MAVE and its discordance with VEP predictions on the scale of full protein sequences. In Figure 4; MAVEs from all 5 categories demonstrated significant correlations with protein attributes. Some differences were observed for annotated regions in proteins where multiple types of assays were performed. For example, 11 residues annotated as a phosphopeptide binding site in CHEK2 had a significant negative difference score from a fitness assay, but a significant positive difference score from an activity assay. Another example is the "non cytoplasmic domain" annotation in KCNE1 which had significant positive z-score differences in the trafficking assay, but not for the activity assay. Differences in discordance driven by MAVE methodology are most evident at the level of specific mutations, such as those seen at important functional residues in proteins assessed with VAMP-seq or other assays not focussed on assessing protein function."*

Minor comments

> *6. Circularity and population bias. The manuscript states that selected population-tuned VEPs are immune or highly resistant to circularity.*
>
> *6.1. Please add citations for this sentence in particular: "We previously found each of these methods to be either immune or highly resistant to the phenomenon of data circularity, so making comparisons using annotated disease variants should not materially affect the results".*

Response: This particular sentence is no longer present in the manuscript due to the additions to the VEP dataset, and we no longer discuss population-tuned VEPs.

> *6.2. I would be interested to see if MAVEs fare better than population-tuned VEPs if the gnomad benign set were derived solely from individuals of non-European ancestry.*

Response: We feel that this is somewhat beyond the scope of this study, although we do note this preprint from our group regarding ancestry bias and predictor performance:

https://www.biorxiv.org/content/10.1101/2024.05.20.594987v3

> *6.3. Please clarify whether gnomAD neuro and cancer cohorts are excluded when constructing the putatively benign set representing the generally "healthy" population.*

Response: We used gnomAD version 4, which, unlike the v3 and v2 releases has no subsets that exclude specific cohorts. From the v4 release blog:

We have removed support for subsets from the gnomAD v4 exomes for two major reasons: given the large size of gnomAD v4 and the small size of any single cohort, we do not feel that the prevalence of any disease is high enough to warrant subsets. In addition, there is increased inclusion of biobank samples, and we do not have concise phenotype metadata for these and many other gnomAD samples, so we cannot ensure that samples in a non-disease subset do not have the specified disease.

> *7. Disorder definition and alternatives.*
>
> *7.1. Please, either justify the use of pLDDT with citations or complement it with an orthogonal IDR caller such as IUPred or Metapredict.*

Response: We have switched from using pLDDT to using a specific intrinsic disorder predictor, AIUPred.

7.2. I would also like to see the counts and effect sizes within predicted IDRs compared to ordered regions. Are MAVEs identifying the handful of variants in IDRs that are truly deleterious or do their scores on average skew deleterious in IDRs?

Response: The counts and effect sizes for annotated disordered regions are provided in Supplementary data 5. In addition, we have clarified this point in the manuscript, noting that both MAVEs and VEPs predominantly assign low-impact scores to variants in intrinsically disordered regions, with MAVEs showing a relative shift toward higher scores compared to VEPs. We also discuss this further in the text:

"VEP performance is known to be reduced in intrinsically disordered regions51,52. These regions are typically less evolutionarily conserved than structured regions, and evolutionary conservation is a key feature in most VEPs (and in some cases the primary signal)39,53. Consistent with this, protein language model predictors such as ESM-1b and ESM-1v show broadly similar trends in intrinsically disordered regions to alignment-based methods such as GEMME, with no clear advantage in IDRs51. As a result, variants in disordered regions tend to be assigned lower impact by VEPs. Although intrinsically disordered regions are enriched in benign variation, both MAVEs and VEPs tend to assign predominantly low-impact scores to variants in these regions, with MAVEs showing a relative shift toward higher scores compared to VEPs. VEPs may therefore under-estimate the impact of variants in these regions due to their reliance on conservation. By contrast, MAVEs are not constrained by evolutionary conservation and can capture context-specific effects in disordered regions. The observed pattern is therefore consistent with VEPs systematically assigning lower impact to variants in disordered regions relative to MAVEs."

7.3. Additionally, it would be useful to individually assess-based and alignment-free VEPs as their performance in IDRs may differ significantly.

Response: We thank the reviewer for this suggestion. We agree that this is an important question, particularly for predictors based on protein language models, which may be viewed as less directly dependent on sequence alignments than classical conservation-based methods. However, our recent analysis of VEP performance in intrinsically disordered regions found little evidence that protein language model predictors behave substantially differently from alignment-based methods in this context (Fawzy & Marsh, 2025). In particular, ESM-based predictors showed similar trends across disordered, intermediate, and ordered regions to alignment-based predictors such as GEMME, with no clear advantage in IDRs. Consistent with this, in the present study we also observe similar relationships between predicted disorder and MAVE/VEP discordance across the VEP groups. We have clarified this point in the revised text.

8. Case examples.

8.1. For SNCA, I would argue that rather than the length of the human lifespan, these mutations would not be seen as pathogenic as their effects would only be seen post-reproductive age, which may be true across other species as well. Further, its to be expected that VEPs trained on conservation across the evolutionary tree would not be able to detect signal from late-onset diseases as well as MAVEs.

Response: We agree that reproductive age, rather than overall lifespan, is the more relevant evolutionary timescale. We have therefore revised the text accordingly. In the updated manuscript, we now note that the pathogenic effect of this variant likely manifests post-reproductively and is therefore not strongly selected against, which may explain the lack of a strong conservation signal.

> *"The exact reason why this assumption does not hold in this case is unclear, but it may be the case that the pathogenic effect of this variant only becomes evident at post-reproductive ages and is thus not strongly selected against."*

8.2. For BRCA1, do all state-of-the-art VEPS beyond the five assessed call the three example mutations as benign? Does concordance for BRCA1 and across all other MAVEs improve if predicted splice variants are filtered out?

Response: We agree that splice-disrupting variants represent an important class that is not captured by most amino acid–focused VEPs and by certain classes of MAVE assays. In the case of BRCA1, the discordant variants we highlight are likely driven by such splice effects, which are detectable in the SGE-based assay but not in cDNA-based assays or most VEPs.

We have not systematically filtered predicted splice-disrupting variants across the full dataset, as this would require the integration of additional models and assumptions, and our aim here is to highlight these discrepancies rather than remove

them. Indeed, such cases illustrate an important limitation of both VEPs and some experimental assays, and are therefore informative for understanding when and why discordance arises.

With the updated VEP set, all VEPs tested except SNPred find the splice-site variants to be non-damaging. We note in the text:

> *"The reason for SNPred identifying the likely splice variants is unclear, as it was not trained with any splicing data, although it was evaluated by its authors using the SGE BRCA1 dataset31."*

> *8.3. For SHOC2, where are the pathogenic mutations, other than S2G, in the protein sequence? Arguably, alignment-based methods will perform poorly at assessing mutations at the beginning or end of a protein sequence as alignment coverage is poor in these regions, rather than being a consequence of the VEPs failing to identify a rare gain-of-function mutation.*

Response: SHOC2 was removed from the dataset after failing the exclusion criteria.

> *9. Minor figure issues and requests.*
>
> *9.1. Add uncertainty and formal tests wherever differences are reported. Bar charts should include error bars or confidence intervals. Show the distribution of Spearman correlations per VEP and per MAVE rather than only means. Include VEP versus VEP and MAVE versus MAVE disagreement maps to show internal dispersion.*

Response: We have revised the analyses to include formal statistical testing throughout, using Wilcoxon rank-sum tests with Bonferroni correction where appropriate. We have also replaced bar charts with boxplots and correlation plots to better represent the underlying distributions, and now report the full distributions of Spearman correlations in the supplementary data files, rather than only summary statistics.

Regarding the suggestion to include detailed VEP-VEP and MAVE-MAVE disagreement maps, we agree that these would be informative for understanding within-method variability. However, our primary aim in this study is to characterise systematic differences between computational predictions and experimental measurements. As VEPs show strong agreement with one another relative to MAVEs, and MAVEs are highly heterogeneous by design, a detailed analysis of within-group disagreement would substantially expand the scope of the work without directly addressing the central question.

> *9.2. Please add residue classification (i.e. hydrophobicity) to the marginals of Figure 3.*

Response: Figure 3 and the supplemental heatmaps have all had their axes ticks colour coded by residue category, and an additional colour key added to the figure.

> *9.3. For RSA and pLDDT differences in Figure 4, please add error bars or CIs to the bar charts if applicable and statistics to see if differences between the concordance classes are significant.*

Response: This section and figure have been completely re-worked. Differences are now displayed on a per-assay basis and measured by Spearman's correlation across the whole score set. The corrected significance threshold is displayed on the figure. P-values are provided in the supplemental datasets.

> *9.4. For Figure 6, please add the distributions to the marginals for each clinical label class and gene.*

Response: This figure is now Figure S7. We re-worked the figure to display all of the data rather than just a few selected proteins. This makes displaying distributions on a per-gene basis unrealistic, but we added density plots for the clinical labels.

> *9.5. Figure S1, please use a perceptually uniform colorway instead of Jet (https://gorelik.net/2020/08/17/what-is-the-biggest-problem-of-the-jet-and-rainbow-color-maps-and-why-is-it-not-as-evil-as-i-thought).*

Response: The colourmap in Figure S1 has been changed to Viridis.

> *9.6. Expansion on Figure S1 – what are the distributions of Spearman's across for each VEP across all MAVEs?*

Response: We have added this as a supplementary dataset (2) and reference it in the text along with Figure S1.

"VEPs of all types tend to rely, to varying degrees, on shared sources of information, particularly evolutionary constraints, and as a result often show substantial agreement with one another41. Consistent with this, the VEPs selected for this study are strongly correlated (Figure S1 and Supplementary Data 2), with a mean Spearman's ρ of 0.84."

Reviewer #3 (Remarks to the Author):

Summary:

Livesey and Marsh ask why variant effect predictors (VEPs) and multiplexed assays of variant effect (MAVEs) differ and offer insights through analysis across 37 MAVEs and 5 state-of-the-art VEPs. This question is extremely important and timely as new recommendations for the classification of genetic variants are being drafted by ClinGen, and as the authors note in the introduction, the clinical community is struggling with the question: "Can we really treat functional measurements from a MAVE and functional predictions from a VEP as independent of each other if they are both attempting to estimate the same value?" To help answer this question the authors detect where MAVEs and VEPs disagree and offer explanations for the cause of this discrepancy. For example, MAVEs are enriched for deleterious measurements at disordered regions compared to VEPs and VEPs tend to overcall deleteriousness at buried regions compared to MAVE measurements. These patterns are consistent with individual MAVE analyses and offer a broader look across many assays. The authors point to specific variants with explainable discordance across 9 proteins and conclude that the combination of MAVE and VEP are ultimately poised to better reflect true fitness effects of variants than either method individually.

Major comments:

1. My biggest critique of the manuscript is the use of normalized rank scores for the MAVE datasets. While I agree with the authors that MAVE data presents a challenge for direct comparison with most VEPs due to different scoring methods and scales, the normalized rank scores compress assay dynamic range and cause wild type-like/functionally normal variants to score like deleterious variants. While this normalization should not impact AUROC values in Figure 1, it can influence each of the subsequent analyses. The problem with ranked scores is that most MAVE scores are wild type-like/functionally normal and assigning ranks to functionally normal variants might artificially cause signal from assay noise. For example, in Figure 2C the MAVE more pathogenic plot might be reflecting wild type-like scores as more pathogenic. We can see from the positional ranked plot in Figure 2A that the region of amino acids 200-250 does not have any variants that rank as high as the positions from amino acids 50-150 and these likely represent functionally normal variants, yet they are called MAVE more pathogenic. A min-max 0-1 normalization should avoid such effects and move many of these variants to the middle of the score distribution while more accurately identifying the rare positions that are MAVE more pathogenic. We can also visualize this effect in the analysis of the MSH2 MAVE from Jia et al. where ~90% of the variants have functionally normal scores and are distributed like synonymous variants. However, ranking variant scores gives many of these wild type-like variants high scores and can have consequences when comparing to VEP outputs, making the MAVE appear to call more variants deleterious and fewer variants wild type-like than is actually reflected in the data. This effect can be visualized in Figure 6 where many of the ClinVar/gnomAD benign MSH2 scores are to the right side of the plot and relatively few are at the top left. Finally, this score normalization also impacts the wide positional score range the authors report for MAVEs. Although this pattern is not unexpected, it can be exaggerated with rank normalization. While the overall patterns that the authors detect are consistent with reported MAVE and VEP concordance/discordance from other studies, the magnitudes of discordance will likely be shifted across many of the genes. This effect might not be as dramatic for VEP score normalization, but the loss of distribution shapes will have an impact on the analysis and I recommend considering a different normalization procedure that maintains distribution shape.

Response: We agree that rank-based normalisation can compress dynamic range and distort the underlying distribution of MAVE scores, particularly for assays in which a large fraction of variants are functionally similar to wild type.

In response, we evaluated several alternative normalisation strategies. As the reviewer suggests, min–max scaling preserves ordering without imposing rank structure, but we found it to be highly sensitive to outliers, particularly for VEPs with long-tailed score distributions (e.g., log-likelihood-based predictors), which led to unstable scaling across datasets.

As a compromise, we have adopted z-score normalisation, which preserves the shape of the underlying score distributions while reducing sensitivity to outliers. This approach avoids the compression effects inherent to rank normalisation and better reflects the relative spread of scores within each dataset. Importantly, the key patterns reported in the manuscript, including the broader positional variance observed in MAVE data, remain consistent under this revised normalisation.

All analyses have been updated accordingly.

2. Additionally, the authors could provide more clarity around assay selection. I thought the introduction to the VEPs and rationale for choosing the five VEPs for comparison was very clear and helpful for setting up and understanding the analysis. However, the MAVE selection was not as clear and as a result, the interpretability of the findings are less clear. For example, the authors could consider breaking down MAVE selection in a similar way to VEPs, but by assay type. MAVEs can be grouped by model system (yeast vs. human cells), by variant integration method (SGE vs. viral integration vs. plasmid), and by functional readout (cell survival vs. protein abundance vs. reporter). Each of these classes has strengths and weaknesses and grouping by class may reveal important distinctions. For example, the authors point out that SGE is typically more accurate than transgene approaches since the SGE cell viability phenotype can be agnostic to variant pathomechanism. However, only 2 SGE datasets are included in the analysis while many more quality SGE datasets have been published in the last few years. For example VHL (PMID: 38969834), TP53 (PMID: 39774325), BRCA2 (PMID: 39779848), DDX3X (PMID: 38057330) and BAP1 (PMID: 38969833) all have accurate SGE datasets which may be important to consider for this analysis. These assays are especially important to consider for the clinical evidence weight problem as SGE assays typically achieve the strongest evidence strength so at minimum, their inclusion in this analysis would be helpful. Ideally, breaking up the analysis by MAVE method would yield the most impactful insights for the clinical community.

Response: As described in our response to Reviewer 2, we have substantially revised our MAVE inclusion criteria. We considered the mentioned SGE datasets in addition to all datasets currently deposited in MaveDB and used in ProteinGym, finding a total of 99 assays across 40 proteins that qualified. We further subset these by broad assay type (activity, fitness, binding, stability or trafficking) and compared these classes to one another throughout the manuscript.

3. Additionally, while the authors provide sound justification for the predictors they choose for the analysis, the two most commonly used predictors in variant classification (REVEL and BayeDel) are left out. Since the rationale for the analysis is built around the use of MAVE and VEP for variant classification, these two should at least be discussed in the main text/discussion. I agree with the authors that these methods are vulnerable to circularity, but some discussion on how this might impact the analysis would also be helpful.

Response: We agree that REVEL and BayesDel are widely used in clinical variant interpretation. However, our aim here was not to include every commonly used predictor, but rather to capture the behaviour of major VEP classes using a representative set of recent, high-performing methods. In the revised manuscript, we have expanded the clinical-trained set to include five predictors, which we believe provides broader coverage of this methodological family than in the original version.

Our focus is on the broad differences between VEPs and MAVEs, rather than on fine-grained distinctions among individual clinical-trained predictors. Given the substantial agreement observed among VEPs within this class, we do not expect inclusion of REVEL or BayesDel to materially alter the main conclusions. We have clarified the rationale for predictor selection in the revised text, and we also now mention these two specifically:

> *"Although some widely used clinical meta-predictors such as REVEL and BayesDel were not included, the expanded clinical-trained set is intended to provide representative coverage of this predictor class."*

Minor comments:

1.Readers likely know this but I suggest adding the word "pooled" in the description of MAVEs on page 2. High throughput could also refer to arrayed experiments that are not technically MAVEs.

Response: We have added the word "pooled" to the description of the MAVEs.

> *"One such approach is to use multiplexed assays of variant effect (MAVEs)3, a general term for a high-throughput, pooled assay in which the effect of a large number of variants on protein function, stability, binding or some other attribute can be assessed."*

2. Typical assay description should add "transfect" after transform to indicate assays in both yeast and human cells.

Response: We have changed the sentence to include "transfect".

> *"In a typical MAVE for a protein coding gene, a library of variants is created in the gene of interest and then used to transform or transfect cells in which the function of the resultant mutant protein is linked to some measurable attribute such as cellular growth rate or reporter gene expression"*

3. This sentence is not technically true since the Pejaver et al paper referenced in the next sentence represents the updated ClinGen recommendation: "Currently VEPs are only capable of providing supporting evidence for pathogenicity while functional assays such as MAVEs can provide up to strong evidence." I think it is fine to change this to "Historically, VEPs were…"

Response: We have updated this text to make the current situation more clear:

> *"Understanding the circumstances in which MAVE and VEP scores are discordant is an important concern for the annotation of variants of uncertain clinical significance (VUS). Both methods are capable of providing complementary evidence under current American College of Medical Genetics and Genomic / Association for Molecular Pathology (ACMG/AMP) guidelines, and can be combined in order to improve evidence strengths15. Under these guidelines, VEPs provide at most supporting evidence for pathogenicity, whereas functional assays such as MAVEs can provide up to strong evidence. However, recent recommendations from the ClinGen Sequence Variant Interpretation working group suggest that, under appropriate calibration, VEPs may provide stronger levels of evidence16."*

4. Figure 3 legend typo: "MAVEs fund" to "MAVEs find".

Response: Typo has been corrected

5. Use of "fitness assay" to describe VAMPseq on page 9 and Table 1 is confusing since VAMPseq is not technically a cellular fitness assay. I suggest just using protein abundance/stability instead.

Response: This has been resolved along with the segregation of MAVEs into the five categories. Specific MAVEs are now referred to by their correct category when referenced in the paper.

Response to reviewers.

Reviewer #3 (Remarks to the Author):

In their revised submission, Livesey and Marsh made significant updates to the analysis and description of results in their manuscript. Updates include broadening both the VEPs (to the top 5 performing population aware and top 5 population free models) and the MAVEs (99 datasets across 47 MAVEs with principled inclusion criteria) offering a more robust view of the agreement and disagreement between the two sources of data. The authors also group MAVEs by type of selection which offers more insight into which methods may agree more than others. In addition, the normalization of VEP and MAVE scores were adjusted to z-score normalization which maintains distribution shape better than rank normalization. Some new compelling results include: patterns of agreement and disagreement are broadly consistent across assay types, suggesting significant overlap in the pathomechanisms detectable by MAVEs. Additionally, variants in DNA-binding domains appear to be consistently over-predicted as deleterious across VEPs highlighting a potential area of consistent bias across model training. These changes satisfy my original concerns about the analysis and add greater nuance to the results and discussion that will be of value to the field and should be carefully considered by groups developing frameworks for the combination of these data sources for clinical variant classification. I have several minor comments to be considered before publication:

We would like to thank reviewer 3 for taking the time to evaluate our work in detail and identify further areas for improvement.

Minor comments:

1) Intro line 94: “40 MAVE datasets” should be 99 scoreset from 47 MAVE studies, or simply MAVE data from 40 human proteins.

We have changed the line to read: “MAVE data from 40 human proteins”.

2) Line 141: “Although some widely used clinical meta-predictors such as REVEL and BayesDel were not included, the expanded clinical-trained set is intended to provide representative coverage of this predictor class.” After reading the author’s response to reviews and the additional analysis of within class VEP differences, I agree with the rationale of excluding these predictors. However, I do think this sentence is out of place and does not offer readers the same context that we have as reviewers. As the authors mention in their response: “Our focus is on the broad differences between VEPs and MAVEs, rather than on fine-grained distinctions among individual clinical-trained predictors. Given the substantial agreement observed among VEPs within this class, we do not expect inclusion of REVEL or BayesDel to materially alter the main conclusions.” I think it would be better to move the description of the exclusion of REVEL and BayesDel to the paragraph starting at line 159 to give the readers the full context of their exclusion.

We have moved the discussion of REVEL and BayesDel to the later paragraph as suggested.

3) Line 173: The separation of MAVEs by type of selection is a great value add to the manuscript. However, the result for performance of fitness based assays is unexpected as SGE in HAP1 cells has been the gold standard for clinical performance amongst MAVEs. Only after looking into the

supplement is it clear that most fitness assays are non-endogenous editing and non-human cell line assays. While I do not have a problem with grouping assays in this way, it would be helpful to the audience to make this distinction within the group since this will be the overwhelming expectation from MAVE and clinical classification scientists. I think this is important to state because it may distract readers from the extremely important conclusion: "This implies a degree of consistency across MAVE datasets despite differences in assay design, with broad patterns of agreement and disagreement with VEPs largely preserved".

Along with the description of the five MAVE categories, we have added a paragraph describing the SGE technology and how the SGE datasets are divided among the activity and fitness assays. We also link Supplementary Data 1 which can be filtered for SGE datasets:

"Saturation genome editing (SGE) is a cutting-edge technology that allows a variant to be engineered into the native gene locus, and subsequently expressed in its native context. In contrast, most other MAVEs overexpress the protein from a plasmid or similar construct. Our dataset contains 8 SGE MAVE datasets for 7 proteins, falling into both the activity and fitness assay categories (Supplementary Data 1). "

4) The SNCA A53T example is confusing. I understand that the low z-score for the population-free VEPs suggests that those predictors consistently call this variant tolerated, but the z-score around zero for the other VEPs and MAVE suggest that they also fail to detect the true pathogenicity of this variant. The interpretation of the z-scores across each dataset in the context of SNCA variants needs to be clarified in this section.

We have clarified that the MAVE and clinical-trained VEPs producing z-scores close to 0 imply a lack of confidence in the prediction rather than a lack of detection.

5) MSH2 S612P is a very interesting example where VEPs may outperform the MAVE, but stating that the ClinVar assessment is likely wrong is a bit of a stretch. This claim should either be backed up by additional evidence (beyond population frequency) or the wording should be hedged significantly. Just as we wouldn't call a variant pathogenic based on a single piece of evidence (in most cases), we should not assume the interpretation is wrong based on the VEP evidence. The VEP evidence is compelling in this case for sure, but since we do not know all of the evidence that Ambry used in this classification, I would stop short of suggesting their interpretation is incorrect.

We no longer directly suggest that the classification is incorrect, and just provide the evidence, leaving the interpretation to the reader.

6) I disagree with the assessment of the CARD11 C49Y variant. While the z-score normalization is a reasonable approach for comparing scores, the interpretation of assay scores would function differently in practice. The MAVE community has adopted language of functionally normal vs functionally abnormal and these distinctions are usually made by the distribution of synonymous variants in the assay. Since the C49Y variant is significantly enriched in the CARD11 fitness assay it would both have a high z-score relative to the synonymous distribution and would be called functionally abnormal. In this sense the assay is actually concordant with the predictors which yield consistent deleterious predictions. This is still a useful example of a caveat to the analysis used in this manuscript, but this would not be considered discordant in variant classification practice. The authors should amend the text to reflect this nuance.

We have changed the end of this paragraph to clarify that both methods are flagging the variant as functionally abnormal, and it is indeed a limitation of this analysis that results in the large z-score difference.

"This is a limitation of this analysis rather than a meaningful difference between the MAVE and VEP scores, as both are flagging the variant as functionally abnormal. The same caveat applies to other gain-of-function variants."
