# Supplementary material for: Why variant effect predictors and multiplexed assays agree and disagree
Source: Nat Commun. 2026 Aug 27;17:10295. doi: 10.1038/s41467-026-77211-x (PMC13620131; doi:10.1038/s41467-026-77211-x)
Supplement: Supplementary file 3 [file 41467_2026_77211_MOESM1_ESM.pdf]

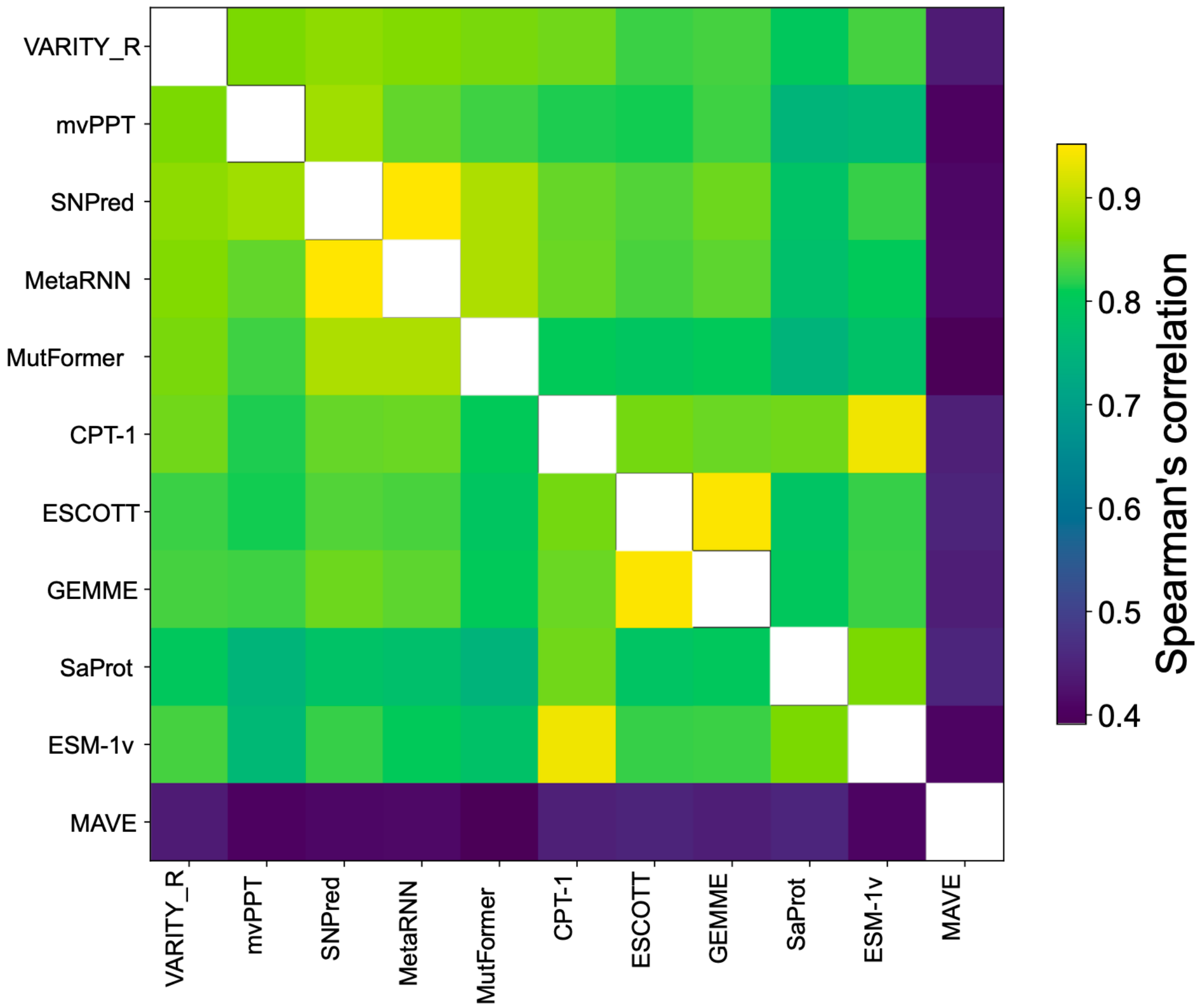

Supplementary Figure 1. Average correlation between VEPs and MAVEs. Overall mean Spearman's correlation (two-sided) between each of the 10 VEPs and the MAVE scores for the selected MAVE assays. Source data for this heatmap are provided in the Source Data file.

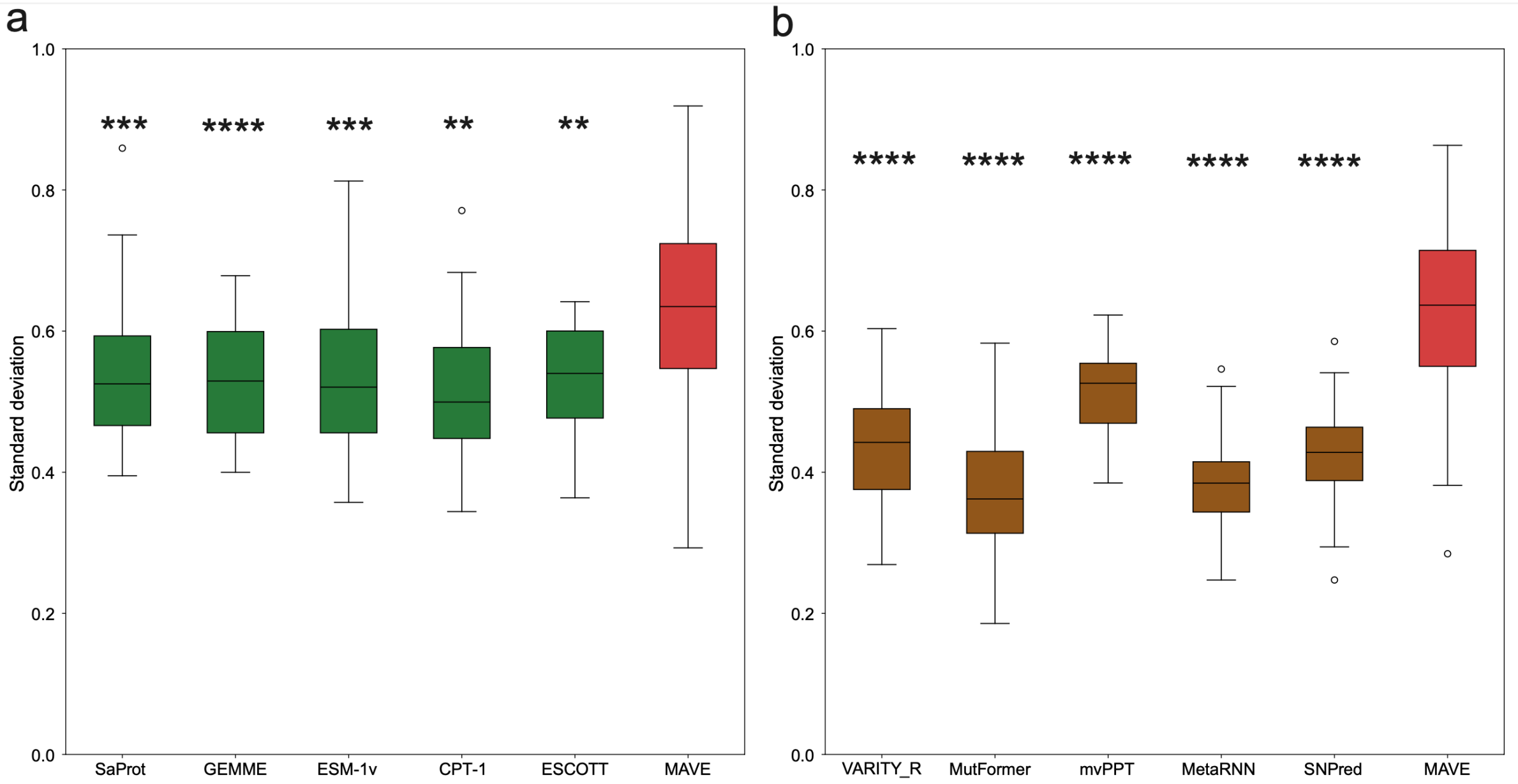

Supplementary Figure 2. Distribution of standard deviations of MAVE and VEP z-scores per protein position. The standard deviation of z-scores at each protein position was pooled across all selected MAVE datasets. Asterisks represent the p-value from the Wilcoxon signed-rank test (two-sided) between the VEP and MAVE distributions for **A)** population-free VEPs (n=40; n=39 for SaProt, CPT and ESCOTT due to missing data) and **B)** clinical-trained VEPs (n=40; n=39 for VARITY_R and n=29 for mvPPT due to missing data). The centre line denotes the median, the box denotes the 25th and 75th percentiles and the whiskers denote the furthest point within 1.5x the interquartile range. Source data for these boxplots are provided in the Source Data file.

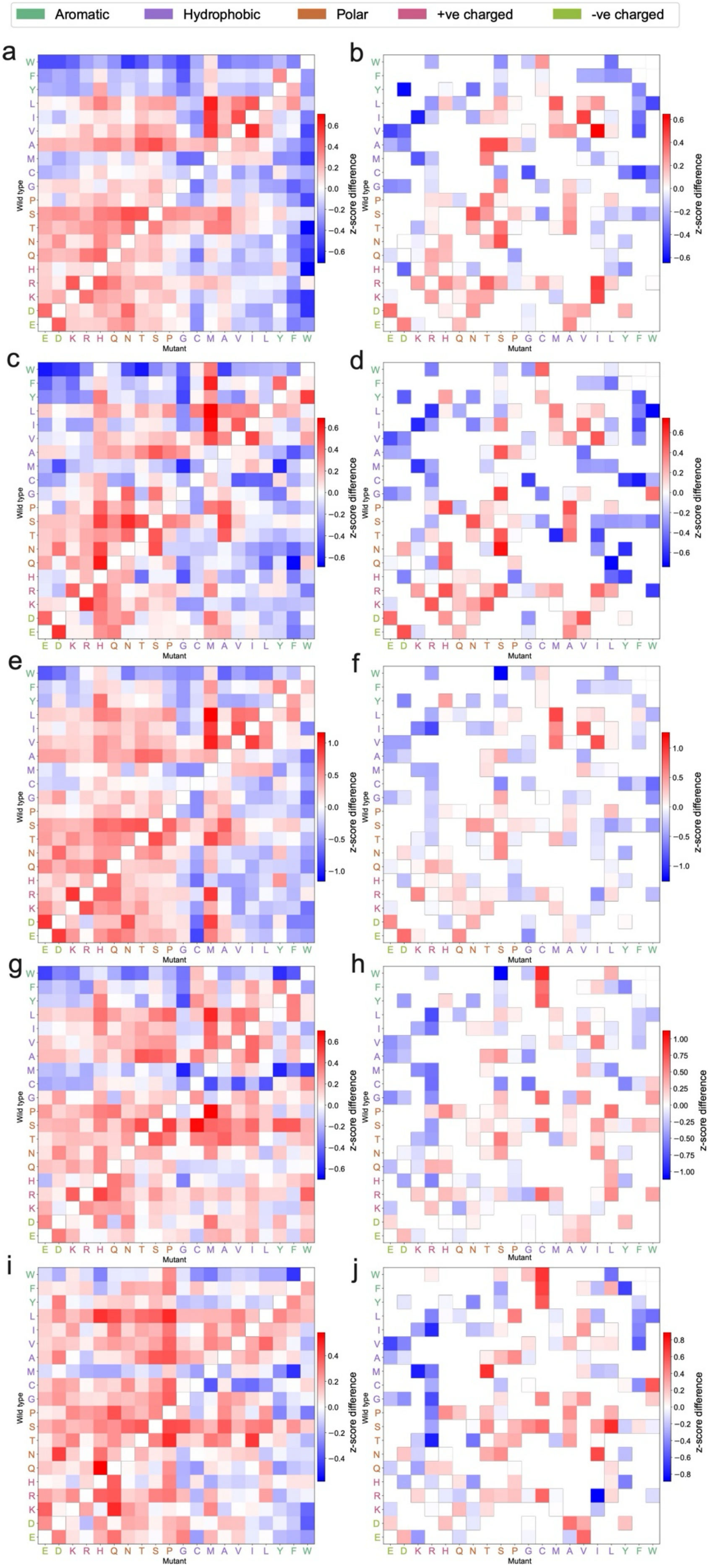

Aromatic
Hydrophobic
Polar
+ve charged
-ve charged
a
b
c
d
e
f
g
h
i
j
Wild type
Mutant
z-score difference
W F Y L I V A M C G P S T N Q H R K D E
E D K R H Q N T S P G C M A V I L Y F W

Supplementary Figure 3. Z-score differences between different MAVE assay selections and VEPs. Heatmaps show the z-score difference between different classes of MAVE assays and VEPs. Positive, red cells indicate substitutions scored as more damaging by MAVEs than by VEPs, while negative, blue cells indicate substitutions scored as more damaging by VEPs than by MAVEs. White squares indicate substitutions for which both methods are concordant. Amino acid properties are indicated by the colour of the axis labels. **A)** Activity-based MAVEs and population-free VEPs. **B)** Activity-based MAVEs and clinical-trained VEPs. **C)** Fitness-based MAVEs and population-free VEPs. **D)** Fitness-based MAVEs and clinical-trained VEPs. **E)** Binding-based MAVEs and population-free VEPs. **F)** Binding-based MAVEs and clinical-trained VEPs. **G)** Stability-based MAVEs and population-free VEPs. **H)** Stability-based MAVEs and clinical-trained VEPs. **I)** Trafficking-based MAVEs and population-free VEPs. **J)** Trafficking-based MAVEs and clinical-trained VEPs. Source data for these heatmaps are provided in the Source Data file.

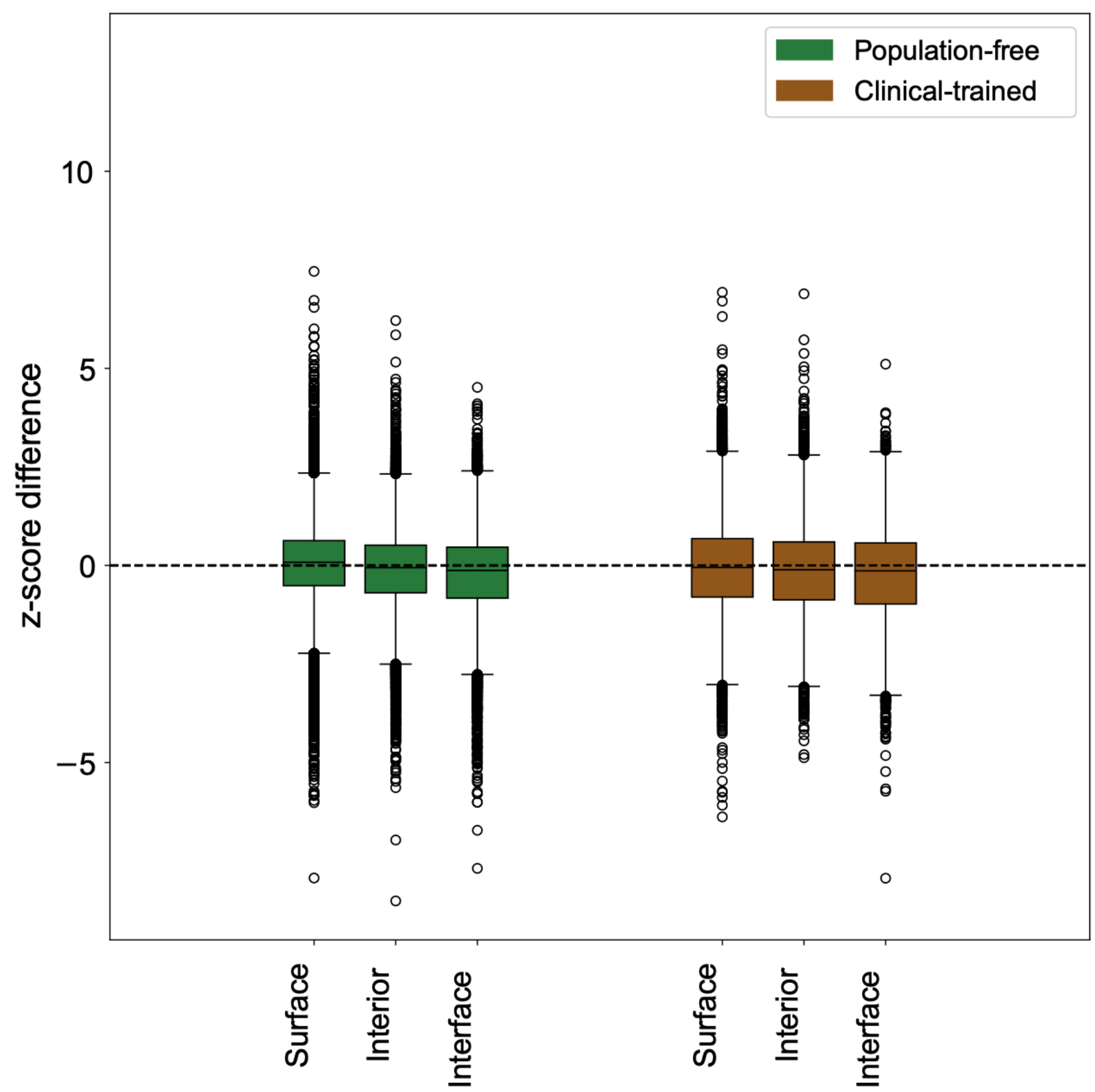

Supplementary Figure 4. Z-score differences between MAVEs and VEPs at the protein surface, interior and interface. Distributions of average z-score differences between the selected MAVE set and VEPs are shown for protein surface, interior and interface residues, for both population-free and clinical-trained predictors. Significance of the differences between structural classes was calculated using the Mann-Whitney U-test (two-sided). For the population-free predictors, n=65,498 for surface, n=83,475 for interior and n=20,726 for interface. For the clinical-trained predictors, n=25,360 for surface, n=32,316 for interior and n=7,946 for interface. The centre line denotes the median, the box denotes the 25th and 75th percentiles and the whiskers denote the furthest point within 1.5x the interquartile range. Source data for these boxplots are provided in the Source Data file.

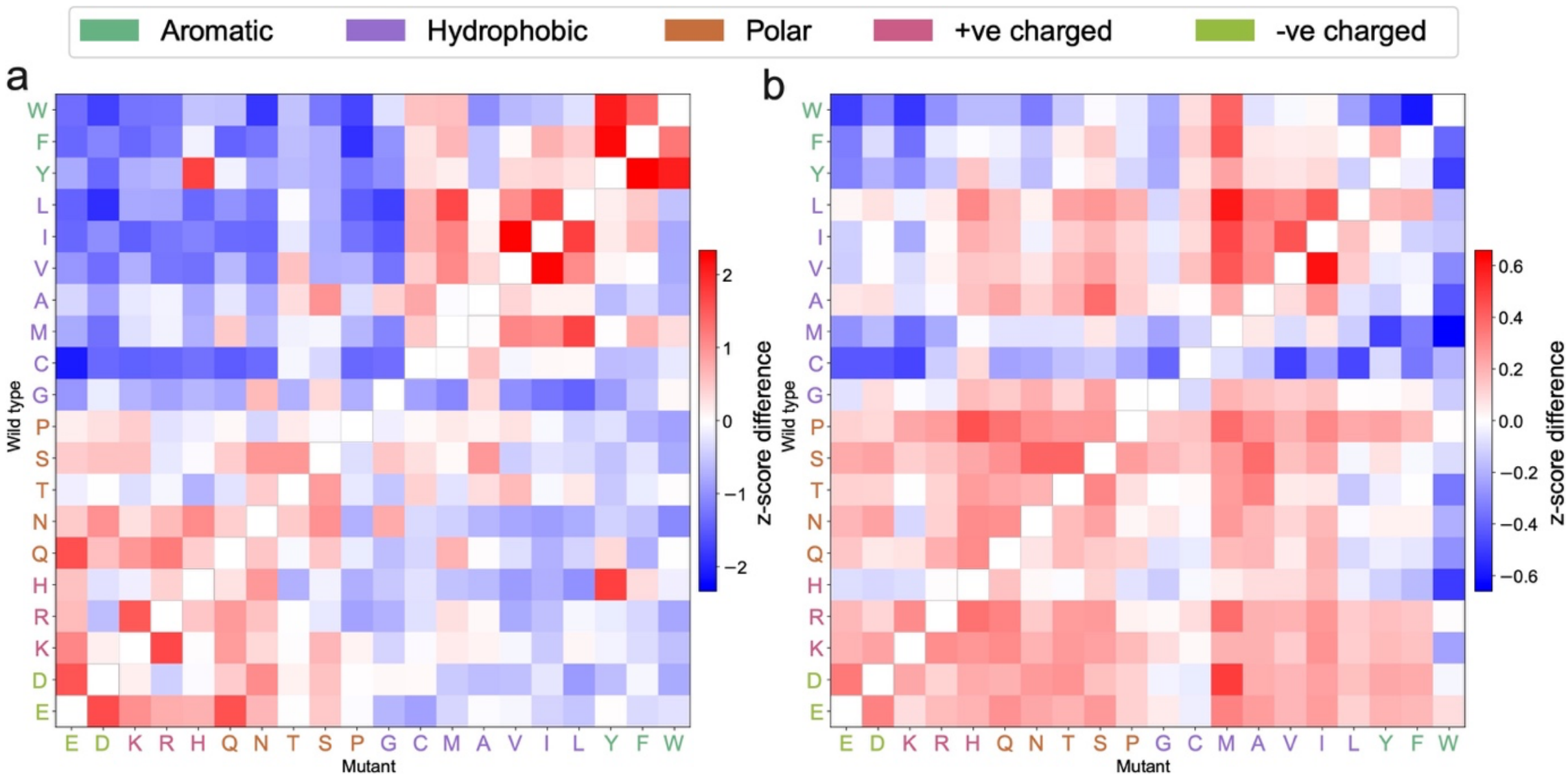

Supplementary Figure 5. Z-score differences between MAVEs, BLOSUM62 and EVE. Heatmaps show the z-score difference between MAVEs, BLOSUM62 and EVE. Positive, red cells indicate substitutions scored as more damaging by MAVEs than by the comparator, while negative, blue cells indicate substitutions scored as more damaging by the comparator than by MAVEs. White squares indicate substitutions for which both methods are concordant. Amino acid properties are indicated by the colour of the axis labels. **A)** MAVEs and the BLOSUM62 substitution matrix. **B)** MAVEs and EVE. Source data for these heatmaps are provided in the Source Data file.

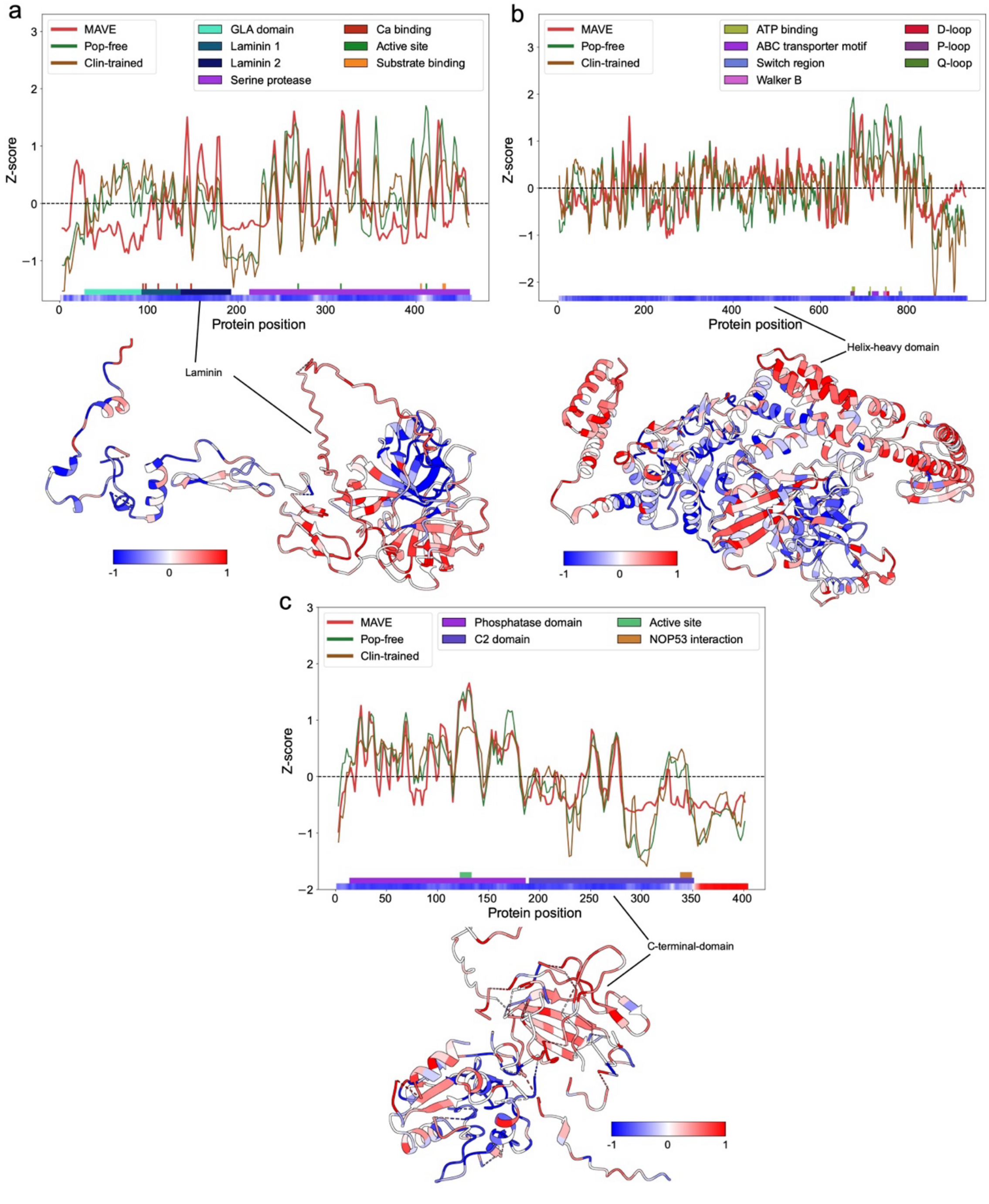

Supplementary Figure 6. Additional examples of z-score difference correspondence with protein features. Moving averages of the MAVE z-scores, population-free VEP z-scores and clinical-trained VEP z-scores within a sliding window of 5 residues are plotted against protein position. Notable protein features and a heatmap representing AIUPred disorder predictions (red indicates disorder) are shown at the bottom of each chart. The difference between the MAVE and population-free VEP z-scores is mapped onto the AlphaFold-predicted protein structure. **A)** F9. **B)** MSH2. **C)** PTEN. Source data for these plots are provided in the Source Data file.

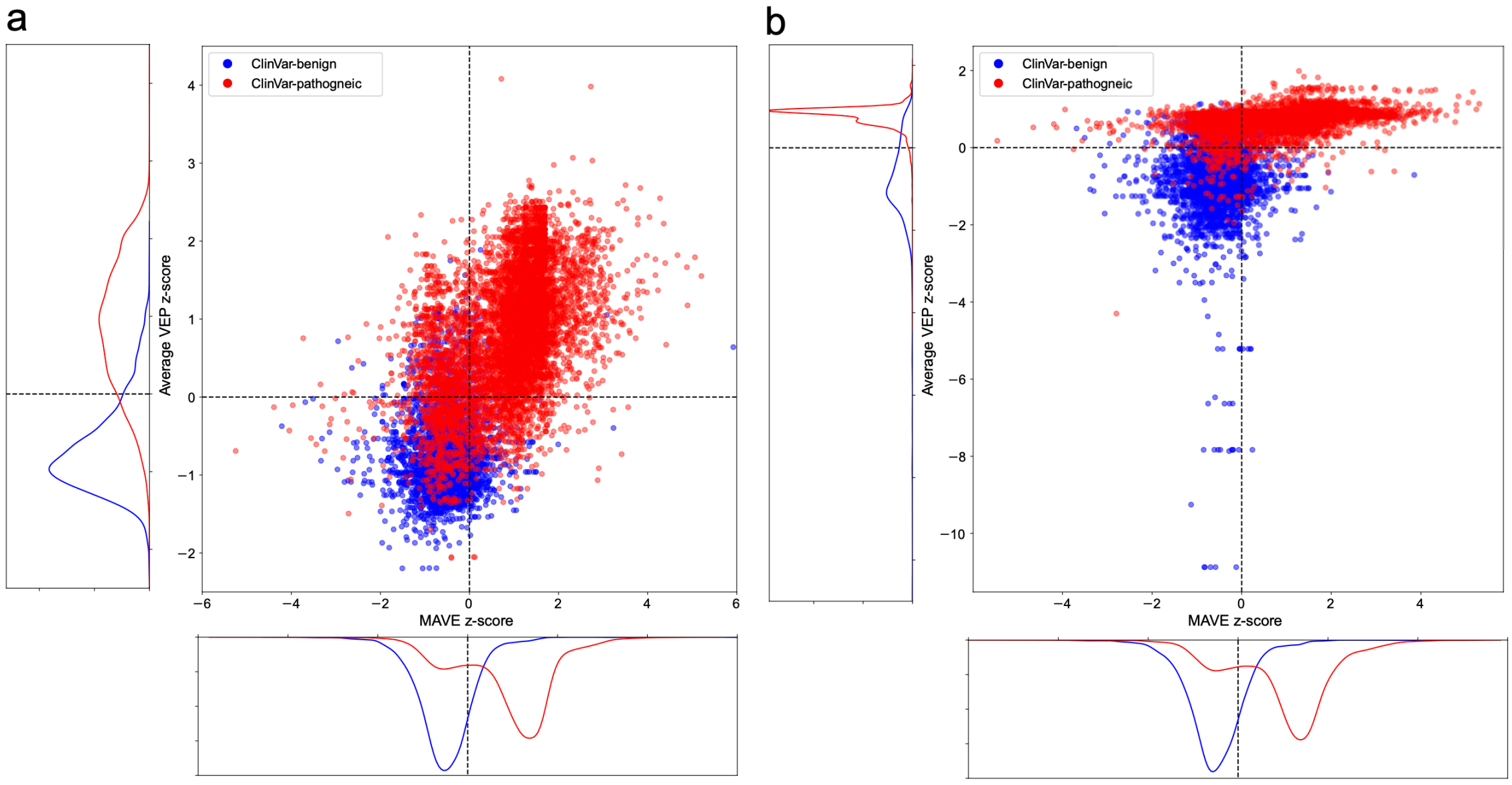

Supplementary Figure 7. Distribution of MAVE and VEP predictions for ClinVar variants. MAVE and VEP z-score distributions are shown for pathogenic and benign variants. **A)** Average population-free VEP z-score plotted against the MAVE z-score for 10,627 comparisons (7,432 pathogenic/likely pathogenic and 3,195 benign/likely benign). **B)** Average clinical-trained VEP z-score plotted against the MAVE z-score for 10,339 comparisons (7,235 pathogenic/likely pathogenic and 3,104 benign/likely benign). Relative densities of VEP z-scores and MAVE z-scores for the pathogenic/likely pathogenic and benign/likely benign groups are shown on each axis. Source data for this plot are provided in the Source Data file.

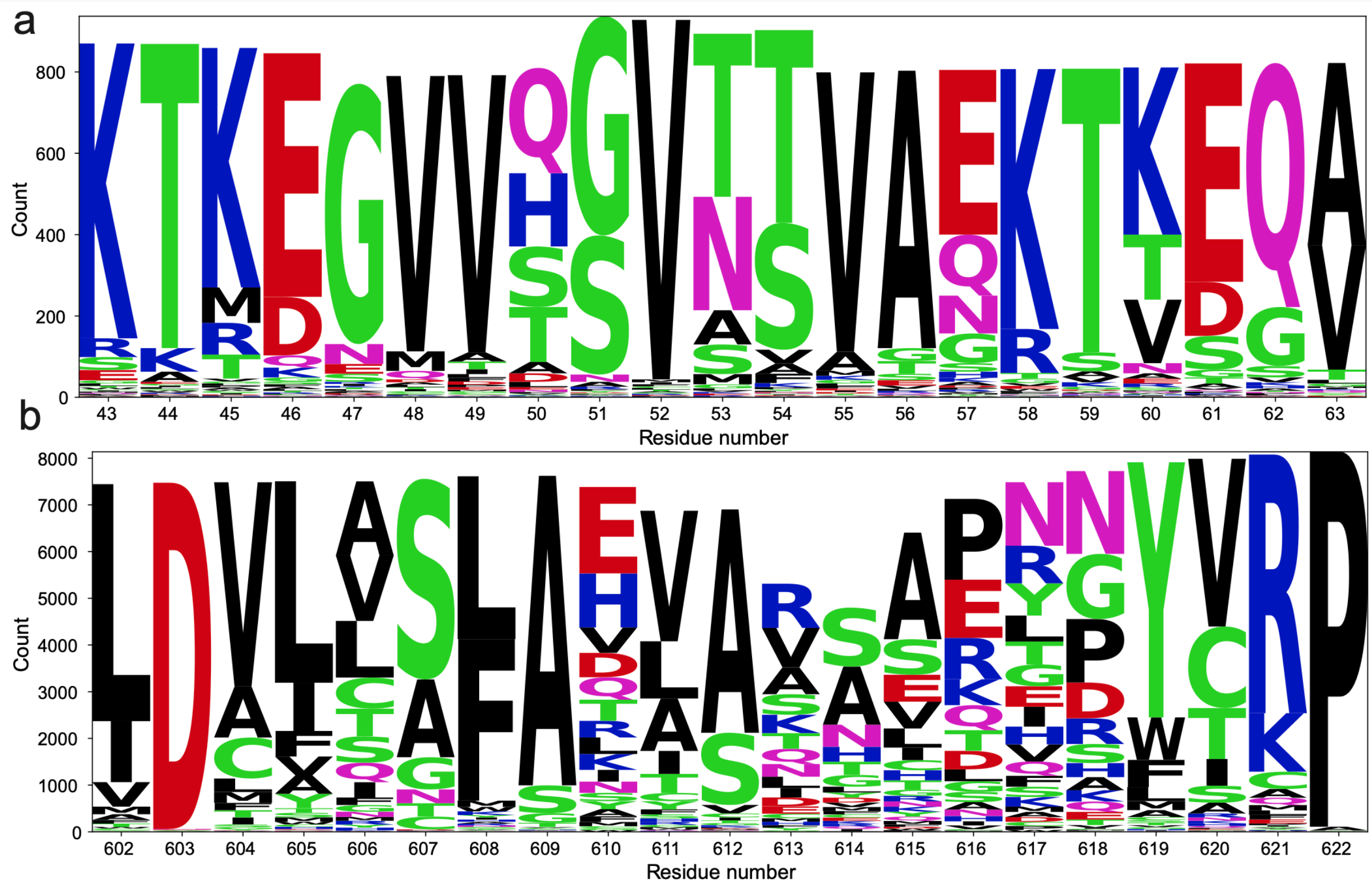

Supplementary Figure 8. Logo plots indicating conservation at ClinVar variants. Multiple sequence alignments were generated using MMseqs2. The size of each letter represents the total number of that amino acid at each aligned position. Differences in column size are due to the removal of gap characters and non-standard amino acid notations from the analysis. **A)** α-synuclein alignment around the pathogenic variant A53T. **B)** MSH2 alignment around the likely benign variant S612P. Source data for these logo plots are provided in the Source Data file.
