# Supplementary material for: Why variant effect predictors and multiplexed assays agree and disagree
Source: Nat Commun. 2026 Aug 27;17:10295. doi: 10.1038/s41467-026-77211-x (PMC13620131; doi:10.1038/s41467-026-77211-x)
Supplement: Supplementary file 4 [file 41467_2026_77211_MOESM2_ESM.pdf]

File Name: Supplementary Data 1
Description: Details of all MAVE assays included in this study.

File Name: Supplementary Data 2
Description: Spearman's correlation between all MAVE assays and the 10 VEPs.

File Name: Supplementary Data 3
Description: Spearman's correlation between MAVE assays performed in the same protein.

File Name: Supplementary Data 4
Description: Z-score difference between population-free and clinical-trained VEPs across amino acid substitutions. Positive scores indicate substitutions identified as more damaging by population-free VEPs.

File Name: Supplementary Data 5
Description: Z-score differences between MAVEs and VEPs within annotated protein features.

File Name: Supplementary Data 6
Description: Z-score differences between population-free and clinical-trained VEPs within annotated protein features. Positive scores indicate features where variants are found more damaging by population-free VEPs.

File Name: Supplementary Data 7
Description: MAVE and VEP Z-scores for pathogenic, likely pathogenic, benign and likely benign variants from ClinVar.

File Name: Supplementary Data 8
Description: AlphaFold and PDB accession identifiers used in this study.

File Name: Supplementary Data 9
Description: Mapping of interface, surface and interior classifications to PDB residues.
